# Risk of subsequent ischemic and hemorrhagic stroke in patients hospitalized for immune-mediated diseases: a nationwide follow-up study from Sweden

**DOI:** 10.1186/1471-2377-12-41

**Published:** 2012-06-18

**Authors:** Bengt Zöller, Xinjun Li, Jan Sundquist, Kristina Sundquist

**Affiliations:** 1 Center for Primary Health Care Research, Lund University/Region Skåne, Clinical Research Centre, Floor 11, Building 28, Entrance 72, Skåne University Hospital, 205 02, Malmö, Sweden; 2 Stanford Prevention Research Centre, Stanford University School of Medicine, Medical School Office Building, 251 Campus Drive, Mail Code 5411, Stanford, California, 94305-5411, USA

## Abstract

**Background:**

Certain immune-mediated diseases (IMDs) have been associated with increased risk for cardiovascular disorders. The aim of the present study was to examine whether there is an association between 32 different IMDs and first hospitalization for ischemic or hemorrhagic stroke.

**Methods:**

All individuals in Sweden hospitalized with a main diagnosis of IMD (without previous or coexisting stroke), between January 1, 1987 and December 31, 2008 (n = 216,291), were followed for first hospitalization for ischemic or hemorrhagic stroke. The reference population was the total population of Sweden. Adjusted standardized incidence ratios (SIRs) for ischemic and hemorrhagic stroke were calculated.

**Results:**

Totally 20 and 15 of the 32 IMDs studied, respectively, were associated with an increased risk of ischemic and hemorrhagic stroke during the follow-up. The overall risks of ischemic and hemorrhagic stroke during the first year after hospitalization for IMD were 2.02 (95% CI 1.90–2.14) and 2.65 (95% CI 2.27–3.08), respectively. The overall risk of ischemic or hemorrhagic stroke decreased over time, to 1.50 (95% CI 1.46–1.55) and 1.83 (95% CI 1.69–1.98), respectively, after 1–5 years, and 1.29 (95% CI 1.23–1.35) and 1.47 (95% CI 1.31–1.65), respectively, after 10+ years. The risk of hemorrhagic stroke was ≥2 during the first year after hospitalization for seven IMDs: ankylosing spondylitis (SIR = 8.11), immune thrombocytopenic purpura (SIR = 8.60), polymyalgia rheumatica (SIR = 2.06), psoriasis (SIR = 2.88), rheumatoid arthritis (SIR = 3.27), systemic lupus erythematosus (SIR = 8.65), and Wegener´s granulomatosis (SIR = 5.83). The risk of ischemic stroke was ≥2 during the first year after hospitalization for twelve IMDs: Addison’s disease (SIR = 2.71), Crohn´s disease (SIR = 2.15), Grave´s disease (SIR = 2.15), Hashimoto´s thyroiditis (SIR = 2.99), immune thrombocytopenic purpura (SIR = 2.35), multiple sclerosis (SIR = 3.05), polymyositis/dermatomyositis (SIR = 3.46), rheumatic fever (SIR = 3.91), rheumatoid arthritis (SIR = 2.08), Sjögren’s syndrome (SIR = 2.57), systemic lupus erythematosus (SIR = 2.21), and ulcerative colitis (SIR = 2.15).

**Conclusions:**

Hospitalization for many IMDs is associated with increased risk of ischemic or hemorrhagic stroke. The findings suggest that several IMDs are linked to cerebrovascular disease.

## Background

Ischemic and hemorrhagic stroke are major causes of morbidity and mortality worldwide [1]. During recent years it has become clear that systemic inflammation may enhance atherogenesis [2-4]. Immune-mediated diseases (IMDs) are a heterogenous group of diseases that are characterized by acute or chronic inflammation [2-8]. Some IMDs have been associated with an increased risk for cardiovascular disease [2-8]. IMDs may increase the cardiovascular disease risk through different mechanisms such as autoreactive lymphocytes, autoantibodies, autoantigens, epigenetic mechanisms, and inflammation driving the formation, progression and rupture of atherosclerotic plaques [2-8]. Inflammation may also affect the thrombotic risk by suppressing fibrinolysis, upregulating procoagulants, and downregulating anticoagulants [7]. Thus, certain IMDs such as rheumatoid arthritis (RA) [3,5,6,8-12] and systemic lupus erythematosus (SLE) [3,5,6,8,13-15] have been associated with an increased risk of cardiovascular disease. Enhanced atherogenesis has also been indicated in other IMDs such as Sjögren´s disease [3,5,6,16], systemic vasculitis [3,5], inflammatory bowel disease [3,5,8,17], and psoriasis [8,18]. As a consequence of this, the risk of stroke has been reported to be increased in patients with systemic lupus erythematosus [19] and rheumatoid arthritis [20].

We hypothesized that not only IMDs such as SLE and RA, but also a number of other less well-studied IMDs have an increased risk of cardiovascular disease. More specifically, we aimed at determining whether IMDs increase the risk for hospitalized ischemic or hemorrhagic stroke. In a nationwide follow-up from 1987–2008 we have estimated the risk of hospitalization with stroke in patients hospitalized with 32 different IMDs without previous or coexisting stroke.

## Methods

This study was approved by the Ethics Committee of Lund University, Sweden. Data used in this study contained information on all individuals registered as residents of Sweden [21]. It included individual-level information on age, sex, occupation, geographic region of residence, hospital diagnoses, and dates of hospital admissions in Sweden (1964–2008), as well as date of emigration, and date and cause of death [21]. The dataset was constructed using several national Swedish data registers (reviewed by Rosen and Hakulinen) [22], including, but not limited to, the Swedish National Population and Housing Census (1960–1990), the Total Population Register, the Multi-Generation Register, and the Swedish Hospital Discharge Register [23]. The data were released to us from the National Board of Health and Welfare and Statistics Sweden.

Information retrieved from the various registers was linked, at the individual level, via the national 10-digit personal identification number assigned to each resident of Sweden for his or her lifetime. Registration numbers were replaced by serial numbers to preserve anonymity. As well as being used to track all records in the database at the individual level, these serial numbers were used to check that individuals with hospital diagnoses of ischemic or hemorrhagic stroke appeared only once during the follow-up (for the first hospital diagnosis of ischemic or hemorrhagic stroke during the study period).

The follow-up period for analysis of data in the present study started on January 1, 1987 and continued until hospitalization for ischemic or hemorrhagic stroke, death, emigration, or the end of the study period (December 31, 2008). Data for first hospitalization for ischemic or hemorrhagic stroke during the study period were retrieved from the Hospital Discharge Register (1987–2008). This study did not include data for hospital outpatients or patients treated at primary health care centers.

### Predictor variable

The predictor variable was hospitalization for an IMD, diagnosed according to ICD-7, ICD-8, ICD-9, and ICD-10 (Additional file 1 Table S1).

### Outcome variable

Diagnosis of ischemic stroke was based on the 9th, and 10th revisions of the International Classification of Diseases (ICD-9, and ICD-10). Cases of ischemic stroke were identified using the following ICD codes: 433, 434, 435, 437.0, and 437.1 (ICD-9); and I63 (not I636), I65, I66, I67.2, and I67.8 (ICD-10).

Diagnosis of hemorrhagic stroke was also based on ICD-9, and ICD-10. Cases of hemorrhagic stroke were identified using the following ICD codes: 431 and 432 (ICD-9); and I61 and I62 (ICD-10).

### Individual-level variables adjusted for in the model

The individual-level variables were sex, age, time period, geographic region of residence, socioeconomic status (SES), and comorbidity.

Sex: male or female.

Age was divided into 5-year categories. Subjects of all ages were included in the study.

Time period was divided into five time periods in order to allow for adjustment for any change in hospitalization rates over time: 1987–1991, 1992–1996, 1997–2001, 2002–2008.

Geographic region of residence was included as an individual-level variable to adjust for possible differences in hospital admissions for ischemic or hemorrhagic stroke between different geographic regions in Sweden. It was categorized as: 1) large city (city with a population of >200,000 (i.e., Stockholm, Gothenburg, or Malmo); 2) Southern Sweden (both rural and urban); and 3) Northern Sweden (both rural and urban).

Occupation was used as a proxy for SES. We classified each individual’s occupation into one of six categories: 1) blue-collar worker, 2) white-collar worker, 3) professional, 4) self-employed, 5) farmer, and 6) non-employed (Individuals without paid employment). Homemakers and students without an occupation were categorized on the basis of their husband’s, father’s or mother’s occupation. If that was not possible, they were included in the “non-employed” category. For individuals aged <20 years, parental occupation was used.

Comorbidity was defined as the first hospital diagnosis at follow up (1987–2008) of the following: 1) chronic lower respiratory diseases (490–496 (ICD-9), and J40–J49 (ICD-10)); 2) obesity (278A (ICD-9), and E65–E68 (ICD-10)); 3) alcoholism and alcohol-related liver disease (291 and 303 (ICD-9), and F10 and K70 (ICD-10)); 4) type 2 diabetes mellitus (250 (age >29 years) (ICD-9), and E11-E14 (ICD-10)); 5) hypertension (401–405 (ICD-9), and I10–I15 (ICD-10)); 6) atrial fibrillation (427D (ICD-9), and I48 (ICD-10)); 7) heart failure (428 (ICD-9), and I50 (ICD-10)); 8) renal disease (580–591 and 753B (ICD-9), and N00-N19, Q61 (ICD-10)); 9) sepsis (036,038 (ICD-9), and A39-A41 (ICD-10)); and 10) coronary heart disease (410–414 (ICD-9), and I20-I25 (ICD-10)).

### Statistical analysis

Person-years at risk (i.e., number of persons at risk multiplied by time at risk) were calculated from the time at which subjects were included in the study (in 1987 or later) until first hospitalization for ischemic or hemorrhagic stroke, death, emigration, or the end of the study period. Person years for IMD patients were calculated from discharge of first hospitalization for IMD (IMD patients with previous stroke before the first IMD hospitalization or at the same hospitalization as the first IMD hospitalization, were excluded). The expected number of cases was based on the number of cases in the reference group. SIRs were calculated as the ratio of observed (O) and expected (E) number of ischemic or hemorrhagic stroke cases using the indirect standardization method [24]:

(1) $$SIR=\frac{\sum_{j-1}^{J}o_{j}}{\sum_{j-1}^{J}n_{j}\lambda_{j}^{*}}=\frac{o}{E^{*}}\text{,}$$

Where $o=\sum o_{j}$ denotes the total observed number of cases in the study group; $E^{*}$ (expected number of cases) is calculated by applying stratum-specific standard incidence rates $\left(\lambda_{j}^{*}\right)$ obtained from the reference group to the stratum-specific person-years (*n*) of risk for the study group; ${{}^{o}}_{j}$ represents the observed number of cases that the cohort subjects contribute to the jth stratum; and J represents the strata defined by cross-classification of the following adjustment variables: age, sex, time period, SES, geographic region of residence, and comorbidity [24]. Ninety-five percent confidence intervals (95% CIs) were calculated assuming a Poisson distribution [24]. All analyses were performed using SAS version 9.2 (SAS Institute, Cary, NC, USA).

## Results

Table 1 shows the number of people admitted to hospital with each of the selected IMDs during the study period. IMD patients with previous stroke before first hospitalization for IMD or stroke at the same time as first IMD hospitalization were excluded from Table 1. Totally 8113 IMD patient with previous or coexisting ischemic stroke and 1416 with hemorrhagic stroke were excluded. A total of 216,291 individuals were hospitalized with an IMD (82,258 males and 134,033 females) (Table 1). The three most common immune-mediated diseases were rheumatoid arthritis (44,611 cases), ulcerative colitis (23,610), and Graves’ disease (22,062). Totally 66,509 patients with ischemic stroke and 428,031 patients with hemorrhagic strokes from 1987–2008 were included (Table 2), of whom 10,905 (9,437 ischemic and 1,468 hemorrhagic strokes) were subsequently admitted to hospital after a first hospitalization for IMD (Table 2). The comorbidities (defined as main or second hospital diagnosis) adjusted for are presented in Table 2.

**Table 1:** Number of cases hospitalizations of IMD and related conditions, 1987-2008

	**Men**		**Women**		**All**	
**Immune-mediated disease**	No	%	No	%	No	%
Addison disease	862	1.05	1190	0.89	2052	0.95
Amyotrophic lateral sclerosis	2376	2.89	2055	1.53	4431	2.05
Ankylosing spondylitis	2416	2.94	1061	0.79	3477	1.61
Autoimmune hemolytic anemia	312	0.38	391	0.29	703	0.33
Behcet disease	146	0.18	138	0.10	284	0.13
Celiac disease	2639	3.21	4249	3.17	6888	3.18
Chorea minor	10	0.01	25	0.02	35	0.02
Crohn disease	9522	11.58	10700	7.98	20222	9.35
Diabetes mellitus type I	9068	11.02	7664	5.72	16732	7.74
Discoid lupus erythematosus	54	0.07	200	0.15	254	0.12
Grave disease	3764	4.58	18298	13.65	22062	10.20
Hashimoto thyroiditis	1440	1.75	5115	3.82	6555	3.03
Immune thrombocytopenic purpura	1905	2.32	2039	1.52	3944	1.82
Localized scleroderma	90	0.11	422	0.31	512	0.24
Lupoid hepatitis	115	0.14	274	0.20	389	0.18
Multiple sclerosis	3492	4.25	6892	5.14	10384	4.80
Myasthenia gravis	935	1.14	1149	0.86	2084	0.96
Pernicious anemia	1663	2.02	1868	1.39	3531	1.63
Polyarteritis nodosa	437	0.53	386	0.29	823	0.38
Polymyalgia rheumatica	5313	6.46	11183	8.34	16496	7.63
Polymyositis/dermatomyositis	404	0.49	667	0.50	1071	0.50
Primary biliary cirrhosis	124	0.15	675	0.50	799	0.37
Psoriasis	4471	5.44	4558	3.40	9029	4.17
Reiter disease	280	0.34	58	0.04	338	0.16
Rheumatic fever	236	0.29	228	0.17	464	0.21
Rheumatoid arthritis	12080	14.69	32531	24.27	44611	20.63
Sarcoidosis	2847	3.46	2518	1.88	5365	2.48
Sjören syndrome	125	0.15	1175	0.88	1300	0.60
Systemic lupus erythematosus	742	0.90	3437	2.56	4179	1.93
Systemic sclerosis	402	0.49	1356	1.01	1758	0.81
Ulcerative colitis	12963	15.76	10647	7.94	23610	10.92
Wegener granulomatosis	1025	1.25	884	0.66	1909	0.88
All	82258	100.00	134033	100.00	216291	100.00

**Table 2:** Number of cases of stroke, 1987-2008

	**All stroke events**					**Subsequent stroke events of auto immune disorders patients**		
	Hemorrhagic stroke		Ischeamic stroke		Hemorrhagic stroke		Ischeamic stroke	
Characteristics	No.	%	No.	%	No.	%	No.	%
Gender								
Men	36639	55.1	216802	50.7	566	38.6	3216	34.1
Women	29870	44.9	211229	49.3	902	61.4	6221	65.9
Age at diagnosis (yrs)								
<50	5070	7.6	13632	3.2	73	5.0	221	2.3
50-59	6857	10.3	29285	6.8	120	8.2	439	4.7
60-69	12399	18.6	71089	16.6	249	17.0	1263	13.4
70-79	21079	31.7	145288	33.9	476	32.4	3208	34.0
> = 80	21104	31.7	168737	39.4	550	37.5	4306	45.6
Period of diagnosis (yrs)								
1987-91	13074	19.7	90088	21.0	134	9.1	832	8.8
1992-96	15114	22.7	110525	25.8	299	20.4	2183	23.1
1997-01	15773	23.7	97993	22.9	389	26.5	2626	27.8
2002-08	22548	33.9	129425	30.2	646	44.0	3796	40.2
Socioeconomic status								
Farmers	4797	7.2	34375	8.0	109	7.4	773	8.2
Self-employed	4911	7.4	31497	7.4	95	6.5	654	6.9
Professionals	4726	7.1	25561	6.0	70	4.8	438	4.6
White collar workers	18912	28.4	119320	27.9	476	32.4	2773	29.4
Workers	29487	44.3	197833	46.2	653	44.5	4406	46.7
Others	3676	5.5	19445	4.5	65	4.4	393	4.2
Region of residence								
Big cities	22409	33.7	145119	33.9	429	29.2	2915	30.9
Northern Sweden	12936	19.4	80680	18.8	310	21.1	1972	20.9
Southern Sweden	31164	46.9	202232	47.2	729	49.7	4550	48.2
Hospitalization for obesity								
Yes	114	0.2	703	0.2	2	0.1	30	0.3
No	66395	99.8	427328	99.8	1466	99.9	9407	99.7
Hospitalization for alcoholism								
Yes	2265	3.4	7448	1.7	45	3.1	143	1.5
No	64244	96.6	420583	98.3	1423	96.9	9294	98.5
Hospitalization for chronic lower respiratory diseases								
Yes	2067	3.1	18287	4.3	63	4.3	613	6.5
No	64442	96.9	409744	95.7	1405	95.7	8824	93.5
Hospitalization for hypertension								
Yes	2254	3.4	16998	4.0	48	3.3	434	4.6
No	64255	96.6	411033	96.0	1420	96.7	9003	95.4
Hospitalization for diabetes type II								
Yes	2425	3.6	28091	6.6	77	5.2	726	7.7
No	64084	96.4	399940	93.4	1391	94.8	8711	92.3
Hospitalization for artrial fibrillation								
Yes	4174	6.3	47257	11.0	134	9.1	1351	14.3
No	62335	93.7	380774	89.0	1334	90.9	8086	85.7
Hospitalization for heart failure								
Yes	4619	6.9	63904	14.9	129	8.8	1749	18.5
No	61890	93.1	364127	85.1	1339	91.2	7688	81.5
Hospitalization for renal disease								
Yes	2656	4.0	19793	4.6	105	7.2	718	7.6
No	63853	96.0	408238	95.4	1363	92.8	8719	92.4
Hospitalization for sepsis								
Yes	2167	3.3	15738	3.7	77	5.2	615	6.5
No	64342	96.7	412293	96.3	1391	94.8	8822	93.5
Hospitalization for coronary heart disease								
Yes	9323	14.0	108895	25.4	234	15.9	2589	27.4
No	57186	86.0	319136	74.6	1234	84.1	6848	72.6
All	66509	100.0	428031	100.0	1468	100.0	9437	100.0

### Hemorrhagic stroke

A total of 66,509 individuals were hospitalized with a main diagnosis of hemorrhagic stroke (Table 2), of whom 1,468 (2·2% of hemorrhagic strokes) had been admitted to hospital due to an IMD (Table 2). The risk of hemorrhagic stroke was significantly increased during the whole follow-up period for 15 of the 32 IMDs studied (Table 3). The overall risk of hemorrhagic stroke during the first year after hospitalization for an IMD was 2·65 (95% CI 2·27–3·08). The overall risk of hemorrhagic stroke decreased over time, to 1·83 after 1–5 years (95% CI 1·69–1·98), 1·63 after 5–10 years (95% CI 1·47–1·80) and 1·47 after 10+ years (95% CI 1·31–1·65).

**Table 3:** SIR for subsequent hemorrhagic stroke of patients with IMD

	**Follow-up interval (years)**																			
	<1				1-5				5-10				> = 10				All			
Immune-mediated diseases	O	SIR	95% CI		O	SIR	95% CI		O	SIR	95% CI		O	SIR	95% CI		O	SIR	95% CI	
Addison´s disease	2	2.70	0.25	9.94	2	0.64	0.06	2.34	1	0.42	0.00	2.41	1	0.55	0.00	3.17	6	0.74	0.27	1.63
Amyotrophic lateral sclerosis	1	0.47	0.00	2.68	2	1.30	0.12	4.78	1	1.82	0.00	10.42	0				4	0.89	0.23	2.30
Ankylosing spondylitis	6	**8.11**	**2.92**	**17.76**	15	**3.43**	**1.92**	**5.68**	7	1.66	0.66	3.44	14	**2.28**	**1.24**	**3.84**	42	**2.72**	**1.96**	**3.67**
Autoimmune hemolytic anemia	1	3.13	0.00	17.91	4	2.96	0.77	7.66	1	1.15	0.00	6.59	2	2.99	0.28	10.98	8	**2.49**	**1.06**	**4.93**
Behcet´s disease	1	33.33	0.01	191.08	0				0				0				1	1.67	0.00	9.55
Celiac disease	2	3.57	0.34	13.13	6	2.08	0.75	4.56	7	**2.69**	**1.07**	**5.58**	8	**2.65**	**1.13**	**5.25**	23	**2.54**	**1.61**	**3.81**
Chorea minor	0				0				0				0				0			
Crohn disease	5	1.47	0.46	3.46	47	**2.60**	**1.91**	**3.46**	19	1.19	0.72	1.87	24	**1.57**	**1.00**	**2.34**	95	**1.80**	**1.46**	**2.21**
Diabetes mellitus type I	0				2	2.50	0.24	9.19	1	1.11	0.00	6.37	2	1.01	0.09	3.70	5	1.32	0.42	3.10
Discoid lupus erythematosus	1	11.11	0.00	63.69	1	2.33	0.00	13.33	0				0				2	1.87	0.18	6.87
Grave´s disease	8	1.53	0.65	3.02	58	**1.77**	**1.35**	**2.29**	48	**1.61**	**1.18**	**2.13**	41	**1.48**	**1.06**	**2.02**	155	**1.62**	**1.38**	**1.90**
Hashimoto´s thyroiditis	4	1.47	0.38	3.79	27	**2.01**	**1.32**	**2.93**	19	**2.10**	**1.26**	**3.29**	11	1.37	0.68	2.46	61	**1.84**	**1.40**	**2.36**
Immune thrombocytopenic purpura	8	**8.60**	**3.67**	**17.03**	12	**2.81**	**1.45**	**4.92**	6	2.18	0.79	4.78	2	1.23	0.12	4.54	28	**2.93**	**1.94**	**4.23**
Localized scleroderma	0				1	0.93	0.00	5.36	1	0.85	0.00	4.90	6	**4.32**	**1.55**	**9.46**	8	2.11	0.90	4.17
Lupoid hepatitis	0				0				0				0				0			
Multiple sclerosis	4	1.82	0.47	4.70	15	1.36	0.76	2.25	9	1.09	0.49	2.08	6	0.94	0.34	2.06	34	1.22	0.84	1.71
Myasthenia gravis	1	1.22	0.00	6.99	8	2.09	0.89	4.14	6	2.17	0.78	4.76	0				15	1.62	0.90	2.68
Pernicious anemia	4	2.15	0.56	5.56	18	1.67	0.99	2.65	13	1.52	0.81	2.61	7	0.99	0.39	2.06	42	**1.49**	**1.07**	**2.01**
Polyarteritis nodosa	2	5.41	0.51	19.88	0	0.00	0.54	2.19	3	1.91	0.36	5.66	0				5	1.00	0.31	2.34
Polymyalgia rheumatica	21	**2.06**	**1.28**	**3.16**	78	**1.42**	**1.12**	**1.77**	65	**1.67**	**1.29**	**2.13**	40	**1.49**	**1.06**	**2.03**	204	**1.56**	**1.35**	**1.79**
Polymyositis/ dermatomyositis	1	2.63	0.00	15.08	3	1.95	0.37	5.77	1	1.18	0.00	6.74	1	2.08	0.00	11.94	6	1.85	0.66	4.04
Primary biliary cirrhosis	1	2.08	0.00	11.94	3	1.76	0.33	5.22	2	2.17	0.20	7.99	0				6	1.87	0.67	4.10
Psoriasis	9	**2.88**	**1.31**	**5.50**	32	**1.83**	**1.25**	**2.59**	23	1.51	0.95	2.26	21	1.32	0.81	2.02	85	**1.64**	**1.31**	**2.03**
Reiter´s disease	0				1	2.94	0.00	16.86	1	2.56	0.00	14.70	0				2	1.42	0.13	5.22
Rheumatic fever	1	7.69	0.00	44.09	0				0				1	1.72	0.00	9.88	2	1.04	0.10	3.81
Rheumatoid arthritis	65	**3.27**	**2.52**	**4.17**	191	**2.03**	**1.76**	**2.34**	109	**1.92**	**1.57**	**2.31**	61	**1.78**	**1.36**	**2.29**	426	**2.08**	**1.89**	**2.29**
Sarcoidosis	3	2.48	0.47	7.34	12	1.87	0.96	3.28	14	**2.26**	**1.23**	**3.80**	6	0.79	0.28	1.73	35	**1.64**	**1.14**	**2.28**
Sjögren´s syndrome	0				3	1.35	0.25	4.00	2	1.03	0.10	3.77	0				5	0.81	0.26	1.90
Systemic lupus erythematosus	9	**8.65**	**3.92**	**16.50**	13	**2.89**	**1.53**	**4.95**	4	1.17	0.31	3.03	6	1.91	0.69	4.19	32	**2.65**	**1.81**	**3.74**
Systemic sclerosis	2	3.17	0.30	11.67	5	2.67	0.84	6.29	3	2.73	0.51	8.07	2	3.45	0.33	12.68	12	**2.87**	**1.48**	**5.03**
Ulcerative colitis	7	1.45	0.57	3.00	40	**1.45**	**1.03**	**1.97**	28	1.21	0.80	1.74	33	1.44	0.99	2.02	108	**1.37**	**1.13**	**1.66**
Wegener´s granulomatosis	6	**5.83**	**2.10**	**12.76**	3	0.90	0.17	2.67	2	1.08	0.10	3.95	0				11	1.47	0.73	2.63
All	175	**2.65**	**2.27**	**3.08**	602	**1.83**	**1.69**	**1.98**	396	**1.63**	**1.47**	**1.80**	295	**1.47**	**1.31**	**1.65**	1468	**1.75**	**1.66**	**1.84**

O = observed number of cases; SIR = standardized incidence ratio; CI = confidence interval.

Bold type: 95% CI does not include 1.00.

Adjusted for age, period, socioeconomic status, region of residence, hospitalization of chronic lower respiratory diseases, obesity, alcoholism, hypertension, diabetes, atrial fibrillation, heart failure, renal disease, sepsis, and coronary heart disease.

The risk of hemorrhagic stroke was ≥2 during the first year after hospitalization for seven IMD (Table 3): ankylosing spondylitis, immune thrombocytopenic purpura, polymyalgia rheumatica, psoriasis, rheumatoid arthritis, systemic lupus erythematosus, and Wegener´s granulomatosis. For seven IMDs, the risk of hemorrhagic stroke was increased 10+ years after hospitalization (Table 3): ankylosing spondylitis, celiac disease, Crohn´s disease, Graves’ disease, localized scleroderma, polymyalgia rheumatica, and rheumatoid arthritis.

### Hemorrhagic stroke and age and sex

The overall risk of hemorrhagic stroke was increased for both sexes at all different follow-up periods (Additional file 1 Tables S2 and S3). The overall risk of hemorrhagic stroke was increased in all age groups for both males and females (<50, 50–59, 60–69, 70–79, and 80+ years) (Additional file 1 Tables S4, S5 and S6).

### Ischemic stroke

A total of 428,031 individuals were hospitalized with a main diagnosis of ischemic stroke (Table 2), of whom 9,437 (2·2% of all ischemic stroke cases) had been admitted to hospital due to an IMD (Table 2). The variables for which the SIRs were adjusted are presented in Table 1. The risk of ischemic stroke was increased during the whole follow-up period for 20 of the 32 IMDs studied (Table 4). The overall risk of ischemic stroke during the first year after hospitalization for an IMD was 2·02 (95% CI 1·90–2·14). The overall risk of ischemic stroke decreased over time, to1·50 after 1–5 years (95% CI 1·46–1·55), 1·38 after 5–10 years (95% CI 1·33–1·43) and 1·29 after 10+ years (95% CI 1·23–1·35) (Table 4).

Ischemic stroke

**Table 4:** SIR for subsequent ischemic stroke of patients with IMD

	**Follow-up interval (years)**																			
	<1				1-5				5-10				> = 10				All			
Immune-mediated diseases	O	SIR	95% CI		O	SIR	95% CI		O	SIR	95% CI		O	SIR	95% CI		O	SIR	95% CI	
Addison´s disease	14	**2.71**	**1.48**	**4.56**	28	1.17	0.78	1.69	30	**1.90**	**1.28**	**2.72**	11	0.97	0.48	1.74	83	**1.48**	**1.18**	**1.83**
Amyotrophic lateral sclerosis	7	0.53	0.21	1.10	16	1.52	0.87	2.47	7	1.77	0.70	3.67	2	1.15	0.11	4.23	32	1.09	0.74	1.54
Ankylosing spondylitis	8	1.62	0.69	3.21	44	**1.55**	**1.13**	**2.08**	24	0.98	0.63	1.46	35	1.08	0.75	1.50	111	**1.23**	**1.01**	**1.48**
Autoimmune hemolytic anemia	4	1.45	0.38	3.75	12	1.00	0.51	1.75	19	**2.51**	**1.51**	**3.93**	5	1.23	0.39	2.90	40	**1.51**	**1.08**	**2.06**
Behcet´s disease	1	4.00	0.00	22.93	1	0.65	0.00	3.70	0				1	1.43	0.00	8.19	3	0.78	0.15	2.29
Celiac disease	9	2.17	0.99	4.14	29	1.28	0.86	1.84	21	1.17	0.72	1.78	26	1.47	0.96	2.15	85	**1.36**	**1.09**	**1.68**
Chorea minor	0				1	2.27	0.00	13.03	0				0				1	1.11	0.00	6.37
Crohn disease	49	**2.15**	**1.59**	**2.84**	160	**1.33**	**1.13**	**1.55**	103	1.11	0.91	1.35	97	1.15	0.93	1.40	409	**1.28**	**1.16**	**1.41**
Diabetes mellitus type I	1	6.25	0.00	35.83	2	0.45	0.04	1.65	5	2.75	0.87	6.46	17	**5.00**	**2.91**	**8.02**	25	**2.54**	**1.64**	**3.76**
Discoid lupus erythematosus	3	4.23	0.80	12.51	3	0.99	0.19	2.92	1	0.47	0.00	2.70	3	1.55	0.29	4.60	10	1.28	0.61	2.37
Grave´s disease	101	**2.15**	**1.76**	**2.62**	402	**1.39**	**1.26**	**1.53**	348	**1.36**	**1.22**	**1.51**	276	**1.27**	**1.12**	**1.43**	1127	**1.39**	**1.31**	**1.48**
Hashimoto´s thyroiditis	77	**2.99**	**2.36**	**3.74**	211	**1.73**	**1.50**	**1.98**	115	**1.39**	**1.14**	**1.67**	82	**1.28**	**1.02**	**1.59**	485	**1.64**	**1.50**	**1.80**
Immune thrombocytopenic purpura	16	**2.35**	**1.34**	**3.83**	55	**1.77**	**1.33**	**2.30**	19	0.94	0.57	1.48	14	1.20	0.65	2.01	104	**1.49**	**1.22**	**1.81**
Localized scleroderma	2	1.28	0.12	4.71	13	1.25	0.66	2.14	18	**1.72**	**1.01**	**2.72**	11	1.04	0.51	1.86	44	1.33	0.97	1.79
Lupoid hepatitis	3	4.48	0.84	13.25	4	1.98	0.52	5.12	0				0				7	2.10	0.83	4.36
Multiple sclerosis	40	**3.05**	**2.18**	**4.15**	73	1.09	0.85	1.37	55	1.11	0.83	1.44	35	0.95	0.66	1.32	203	**1.22**	**1.06**	**1.40**
Myasthenia gravis	6	1.01	0.36	2.21	38	1.36	0.96	1.87	23	1.20	0.76	1.80	13	1.08	0.57	1.85	80	1.23	0.97	1.53
Pernicious anemia	25	**1.56**	**1.01**	**2.31**	138	**1.49**	**1.25**	**1.76**	89	1.23	0.99	1.52	74	**1.44**	**1.13**	**1.80**	326	**1.40**	**1.25**	**1.56**
Polyarteritis nodosa	5	1.23	0.39	2.89	20	1.30	0.79	2.02	11	1.05	0.52	1.88	10	1.13	0.54	2.08	46	1.19	0.87	1.58
Polymyalgia rheumatica	165	**1.76**	**1.50**	**2.05**	761	**1.50**	**1.39**	**1.61**	529	**1.54**	**1.41**	**1.68**	322	**1.53**	**1.37**	**1.71**	1777	**1.54**	**1.47**	**1.61**
Polymyositis/ dermatomyositis	10	**3.46**	**1.65**	**6.39**	13	1.19	0.63	2.03	6	1.07	0.38	2.34	5	1.73	0.55	4.07	34	**1.52**	**1.05**	**2.13**
Primary biliary cirrhosis	4	1.54	0.40	3.98	11	1.45	0.72	2.60	4	0.91	0.24	2.35	1	2.17	0.00	12.46	20	1.33	0.81	2.05
Psoriasis	44	**1.92**	**1.39**	**2.58**	217	**1.65**	**1.44**	**1.89**	163	**1.53**	**1.30**	**1.78**	144	**1.41**	**1.19**	**1.66**	568	**1.56**	**1.44**	**1.70**
Reiter´s disease	0				5	2.02	0.64	4.76	6	2.47	0.89	5.41	2	0.56	0.05	2.07	13	1.47	0.78	2.52
Rheumatic fever	5	**3.91**	**1.23**	**9.19**	10	1.66	0.79	3.06	14	**3.04**	**1.65**	**5.11**	7	1.81	0.72	3.75	36	**2.28**	**1.59**	**3.16**
Rheumatoid arthritis	345	**2.08**	**1.86**	**2.31**	1266	**1.66**	**1.57**	**1.75**	663	**1.45**	**1.34**	**1.56**	326	**1.30**	**1.16**	**1.45**	2600	**1.59**	**1.53**	**1.65**
Sarcoidosis	9	0.97	0.44	1.85	70	**1.43**	**1.12**	**1.81**	51	1.12	0.83	1.47	56	1.08	0.81	1.40	186	**1.19**	**1.03**	**1.38**
Sjögren´s syndrome	10	**2.57**	**1.22**	**4.75**	28	1.38	0.92	1.99	15	0.96	0.54	1.59	15	1.26	0.70	2.08	68	**1.31**	**1.02**	**1.67**
Systemic lupus erythematosus	19	**2.21**	**1.33**	**3.46**	88	**2.33**	**1.87**	**2.87**	54	**1.92**	**1.44**	**2.51**	30	1.26	0.85	1.80	191	**1.94**	**1.68**	**2.24**
Systemic sclerosis	11	1.90	0.94	3.41	28	1.22	0.81	1.77	11	1.19	0.59	2.14	2	0.39	0.04	1.43	52	1.21	0.90	1.58
Ulcerative colitis	71	**2.15**	**1.68**	**2.71**	231	**1.27**	**1.11**	**1.45**	162	1.09	0.92	1.27	146	1.05	0.89	1.24	610	**1.21**	**1.12**	**1.31**
Wegener´s granulomatosis	11	1.66	0.82	2.98	12	0.47	0.24	0.83	26	**1.54**	**1.00**	**2.25**	12	1.69	0.87	2.96	61	1.09	0.83	1.40
All	1075	**2.02**	**1.90**	**2.14**	3990	**1.50**	**1.46**	**1.55**	2592	**1.38**	**1.33**	**1.43**	1780	**1.29**	**1.23**	**1.35**	9437	**1.46**	**1.43**	**1.49**

O = observed number of cases; SIR = standardized incidence ratio; CI = confidence interval.

Bold type: 95% CI does not include 1.00.

Adjusted for age, period, socioeconomic status, region of residence, hospitalization of chronic lower respiratory diseases, obesity, alcoholism, hypertension, diabetes, atrial fibrillation, heart failure, renal disease, sepsis, and coronary heart disease.

The risk of ischemic stroke was ≥2 during the first year after hospitalization for twelve IMDs (Table 4): Addison’s disease, Crohn´s disease, Grave´s disease, Hashimoto´s thyroiditis, immune thrombocytopenic purpura, multiple sclerosis, polymyositis/dermatomyositis, rheumatic fever, rheumatoid arthritis, Sjögren’s syndrome, systemic lupus erythematosus, and ulcerative colitis. For seven IMDs, the risk of ischemic stroke was increased 10+ years after hospitalization: diabetes mellitus type 1, Graves’ disease, Hashimoto’s thyroiditis, pernicious anemia, polymyalgia rheumatica, psoriasis, and rheumatoid arthritis (Table 4).

### Ischemic stroke and age and sex

The overall risk of ischemic or hemorrhagic stroke was increased for both sexes at all different follow-up periods (Additional file 1 Tables S7 and S8). The overall risk of ischemic stroke was increased in all age groups for both sexes (<50, 50–59, 60–69, 70–79, and 80+ years) (Additional file 1 Tables S9, S10 and S11).

### Time period and hemorrhagic and ischemic stroke

The overall risk for both hemorrhagic and ischemic stroke was slightly higher between 1987 and 1996 (1.98 95% CI 1.78-2.20 and 1.51 95% CI 1.45-1.57, respectively) than between 1997 and 2008 (1.58 95% CI 1.48-1.69 and 1.38 95% CI 1.34-1.41, respectively) (Additional file 1 Tables S12 and S13).

## Discussion

The present study is the first nationwide study of IMDs and ischemic and hemorrhagic stroke. The results indicate that several IMDs increase the risk of hospitalization for both ischemic and/or hemorrhagic stroke. The relative risk of ischemic and hemorrhagic stroke during the first year after hospitalization with certain IMDs was even higher than the risks associated with many traditional risk factors for ischemic and hemorrhagic stroke [1,25]. Although it declined over time, the overall risk of ischemic and hemorrhagic stroke remained elevated for 10 or more years for some IMDs. The results of our study are in line with previous studies linking rheumatoid arthritis [3,5,6,8-12,20], systemic lupus erythematosus [3,5,6,8,13-15,19], Sjögren´s disease [3,5,6,16], systemic vasculitis [3,5], inflammatory bowel disease [3,5,8,17], and psoriasis [8,18] to an increased risk of cardiovascular disease. However, what distinguishes our study from these other studies is its comparison of large numbers of patients and 32 types of IMDs with the general population in a nationwide setting, as well as the long-term follow-up of patients and the determination of risk for both ischemic and hemorrhagic stroke. Moreover, we also found a number of novel associations between IMDs and ischemic and hemorrhagic stroke. The results of the present study suggest that increased risk of subsequent ischemic and hemorrhagic stroke is a common feature of several IMDs, not just selected conditions such as systemic lupus erythematosus [19] and rheumatoid arthritis [20].

Although the increased risk of ischemic and hemorrhagic stroke may have different underlying causes in different IMDs, a general link between systemic inflammation and atherothrombosis has been indicated [2-8]. In some conditions, such as in immune thrombocytopenic purpura, hemorrhagic stroke may occur as the direct result of thrombocytopenia. The formation of autoantibodies may, in special cases, also contribute to stroke [26]. The increased risk of stroke may be specific for more severe cases of IMDs, since the patients in our study had been admitted to hospital. The effects of treatment—corticosteroids promote hemostasis [27]—and the effect of inflammation on coagulation [7] may also contribute to the identified associations. Hypothetically, the fact that the risk of ischemic and hemorrhagic stroke decreased over time may suggest that it is linked to the inflammatory activity of the IMDs, which is likely to decrease over time due to treatment. In line with this hypothesis, in several studies disease activity appears to be linked with atherosclerosis progression [2-8,28,29]. However, as we lack treatment data, we cannot prove this hypothesis but in this context it is interesting that the relative risk of both hemorrhagic and ischemic stroke was lower between 1997 and 2008 than between 1987 and 1996 (Additional file 1 Tables S12 and S13).

The present study has certain limitations. For example, we had no data on general cardiovascular disease risk factors such as weight, smoking, and diet. It is unrealistic to gather such data for an entire national population. However, we did adjust for socioeconomic status, which is associated with risk factors such as smoking. Aspirin and non-steroidal anti-inflammatory drugs (NSAID) may affect the risk of ischemic and hemorrhagic stroke [30,31]. However, we had no access to treatment data. Adjustment was, however, made for several comorbidities (chronic lower respiratory diseases, obesity, alcoholism and alcohol-related liver disease, type 2 diabetes mellitus, hypertension, atrial fibrillation, coronary heart diseases, heart failure, renal disease and sepsis). Still, residual bias may remain due to hospitalization of the most severe cases with IMD. However, all cases with previous or coexisting stroke were excluded to avoid selection bias. Totally, 8113 IMD patients with previous or coexisting ischemic stroke and 1416 with hemorrhagic stroke were excluded from the study, which in turn instead may underestimate the stroke risk. In fact, our results are within the limit for published cardiovascular disease risk in IMDs like RA [3,5,6,8-12,20] and SLE [3,5,6,8,13-15,19]. Thus, the estimated risks of stroke in IMD patients appear to be fairly valid. Anyway, the present study reflects the real world risks for stroke among hospitalized IMD (without previous stroke or at the same time as first hospitalization for IMD). All cases of ischemic and hemorrhagic stroke in Sweden should, according to official guidelines, be treated at hospitals [32]. Moreover, hospitalization incidence rates were calculated for the whole follow-up period, divided into five time periods, and adjustments were made for possible changes in hospitalization rates over time.

This study also has a number of strengths. The study reflects the situation in real world medicine during 22 years in a country with a high standard in the medical diagnosis [22,23,33-35]. The study population included all individuals clinically diagnosed with IMD and ischemic and hemorrhagic stroke in hospital during the study period, which eliminated recall bias. Because of the personal identification number assigned to each resident in Sweden, it was possible to trace all subjects for the whole follow-up period. Data on occupation were 99·2% complete (1980 and 1990 censuses), which enabled us to adjust our models for socioeconomic status. A further strength of the present study was the use of validated hospital discharge data. The Hospital Discharge Register has high validity [22,23,33-35], especially for cardiovascular disorders such as stroke, for which approximately 95% of diagnoses have been shown to be correct [33-35]. Though, the positive predictive value (PPV) may differ between diagnoses in the Swedish Hospital Discharge Register, the PPV is generally around 85-95% [35].

## Conclusions

In summary, the risk of hospitalization for ischemic and hemorrhagic stroke was, for several immune-mediated diseases studied, found to be significantly associated. The risk of ischemic and hemorrhagic stroke during the first year after hospitalization with an immune-mediated disease was high for certain IMDs. Although it decreased over time, for some IMDs the risk of ischemic and hemorrhagic stroke remained elevated for more than 10 years. The findings of the present study suggest that many IMDs are linked to cerebrovascular disease. Future studies could elucidate the mechanisms behind stroke in specific IMDs.

## Abbreviations

CI, Confidence interval; E, Expected; ICD, international classification of diseases; IMD, immune-mediated disease; O, Observed; RA, rheumatoid arthritis; SES, socioeconomic status; SIR, standarized incidence ratio; SLE, systemic lupus erythematosus.

## Competing interests

The authors declare that they have no competing interests.

## Authors’ contributions

All authors contributed to the conception and design of the study; JS and KS contributed to the acquisition of data; all authors contributed to the analysis and interpretation of data; BZ drafted the manuscript; and all authors revised it critically and approved the final version. All authors had full access to all of the data (including statistical reports and tables) and take responsibility for the integrity of the data and the accuracy of its analysis.

## Pre-publication history

The pre-publication history for this paper can be accessed here:

http://www.biomedcentral.com/1471-2377/12/41/prepub

## Supplementary Material

Additional file 1 **Table S1**. ICD codes of IMD and related conditions. Table S2. SIR for subsequent hemorrhagic stroke of male patients with IMD. Table S3. SIR for subsequent hemorrhagic stroke of female patients with IMD. Table S4. SIR for subsequent hemorrhagic stroke of patients with IMD after one year of follow-up. Table S5. SIR for subsequent hemorrhagic stroke of male patients with IMD after one year of follow-up. Table S6. SIR for subsequent hemorrhagic stroke of female patients with IMD after one year of follow-up. Table S7. SIR for subsequent ischemic stroke of male patients with IMD. Table S8. SIR for subsequent ischemic stroke of female patients with IMD. Table 9. SIR for subsequent ischemic stroke of patients with IMD. Table S10. SIR for subsequent ischemic stroke of male patients with IMD after one year of follow-up. Table S11. SIR for subsequent ischemic stroke of female patients with IMD after one year of follow-up. Table S12. SIR for subsequent hemorrhagic stroke of patients with IMD after one year of follow-up. Table S13. SIR for subsequent ischemic stroke of patients with IMD after one year of follow-up. Click here for file
