# Supplementary material for: Risk of subsequent ischemic and hemorrhagic stroke in patients hospitalized for immune-mediated diseases: a nationwide follow-up study from Sweden
Source: BMC Neurol. 2012 Jun 18;12:41. doi: 10.1186/1471-2377-12-41 (PMC3430565; doi:10.1186/1471-2377-12-41)
Supplement: Additional file 1 — Table S1. ICD codes of IMD and related conditions. Table S2. SIR for subsequent hemorrhagic stroke of male patients with IMD. Table S3. SIR for subsequent hemorrhagic stroke of female patients with IMD. Table S4. SIR for subsequent hemorrhagic stroke of patients with IMD after one year of follow-up. Table S5. SIR for subsequent hemorrhagic stroke of male patients with IMD after one year of follow-up. Table S6. SIR for subsequent hemorrhagic stroke of female patients with IMD after one year of follow-up. Table S7. SIR for subsequent ischemic stroke of male patients with IMD. Table S8. SIR for subsequent ischemic stroke of female patients with IMD. Table 9. SIR for subsequent ischemic stroke of patients with IMD. Table S10. SIR for subsequent ischemic stroke of male patients with IMD after one year of follow-up. Table S11. SIR for subsequent ischemic stroke of female patients with IMD after one year of follow-up. Table S12. SIR for subsequent hemorrhagic stroke of patients with IMD after one year of follow-up. Table S13. SIR for subsequent ischemic stroke of patients with IMD after one year of follow-up. [file 1471-2377-12-41-S1.doc]

**Supplementary Table 1. ICD codes of IMD** and related conditions

|  | **ICD code** | | | |
| --- | --- | --- | --- | --- |
| **Immune-mediated disease** | **ICD-7** | **ICD-8** | **ICD-9** | **ICD-10** |
| Addison’s disease | 274.4 | 255.1 | 255E | E27.1, E27.2 |
| Amyotrophic lateral sclerosis | 356.1 | 348 | 335C | G12.2 |
| Ankylosing spondylitis | 722.1 | 712.4 | 720A | M45, M08.1 |
| Autoimmune hemolytic anemia | 292.2 | 283.90, 283.92 | 283A | D59.0 |
| Behçet’s disease | - | 136.0 | 136B | M35.2 |
| Celiac disease | 286.00 | 269.00, 269.98 | 579A | K90.0 |
| Chorea minor | 402 | 392.9 | 392X | I02.9 |
| Crohn’s disease | 572.00, 572.09 | 563.00 | 555 | K50 |
| Diabetes mellitus type I | 260 (age <20 yr) | 250 (age <20 yr) | 250 (age <20 yr) | E10 (age <20 yr) |
| Discoid lupus erythematosus | 705.4 | 695.4 | 695E | L93.0 |
| Graves’ disease | 252 | 242 | 242 | E05 |
| Hashimoto’s thyroiditis | 253 | 245.1, 243, 244 | 245C, 243, 244 | E00-E03, E06.3 |
| Immune thrombocytopenic purpura | 296 | 287.3 | 287D | D69.3 |
| Localized scleroderma | - | 701.0 | 701A | L94.0 |
| Lupoid hepatitis | 583 | 573.9 | 571EJ | K75.4 |
| Multiple sclerosis | 345 | 340 | 340 | G35 |
| Myasthenia gravis | 744.0 | 733.0 | 358A | G70.0 |
| Pernicious anemia | 290.0 | 281.0 | 281A | D51.0 |
| Polyarteritis nodosa | - | 446.0 | 446A | M30.0 |
| Polymyalgia rheumatica | - | 717.9 | 725 | M315, M35.3 |
| Polymyositis/dermatomyositis | 710.00, 710.01, 726.30 | 716.0, 716.1 | 710D, 710E | M33 |
| Primary biliary cirrhosis | - | - | 571G | K74.3 |
| Psoriasis | 706 | 696 | 696 | L40 |
| Reiter’s disease | - | 099.3, 711.1 | 099D, 711B | M02.3 |
| Rheumatic fever | 400-401 | 390-392 (not 392.9) | 390-392 (not 392X) | I00-02 (not I02.9) |
| Rheumatoid arthritis | 722 (not 722.1) | 712.1, 712.3 | 714 (not 714E, 714X) | M05, M06, M08.0, M08.2 |
| Sarcoidosis | 138.00-138.10 | 135 | 135 | D86 |
| Sjögren’s syndrome | - | 734.90 | 710C | M35.0 |
| Systemic lupus erythematosus | 705.4 | 734.1 | 710A | M32 |
| Systemic sclerosis | 710.0 (not 710.00, 710.01) | 710.0, 734.0 | 710B | M34 |
| Ulcerative colitis | 572.20, 572.21 | 563.10, 569.02 | 556 | K51 |
| Wegener’s granulomatosis | 456 | 446.3 | 446E | M31.3 |

| **Supplement Table 2. SIR for subsequent hemorrhagic stroke of male patients with IMD** | | | | | | | | | | | | | | | | | | | | | | | | |
| --- | --- | --- | --- | --- | --- | --- | --- | --- | --- | --- | --- | --- | --- | --- | --- | --- | --- | --- | --- | --- | --- | --- | --- | --- |
|  | Follow-up interval (years) | | | | | | | | | | | | | | | | | | |  |  |  |  |  |
|  | <1 | | | |  | 1-5 | | | |  | 5-10 | | | |  | >=10 | | | |  | All | | | |
| Immune-mediated diseases | O | SIR | 95% CI | |  | O | SIR | 95% CI | |  | O | SIR | 95% CI | |  | O | SIR | 95% CI | |  | O | SIR | 95% CI | |
| Addison´s disease | 1 | 2.33 | 0.00 | 13.33 |  | 1 | 0.68 | 0.00 | 3.93 |  | 1 | 0.90 | 0.00 | 5.16 |  | 0 |  |  |  |  | 3 | 0.78 | 0.15 | 2.32 |
| Amyotrophic lateral sclerosis | 1 | 0.81 | 0.00 | 4.66 |  | 0 |  |  |  |  | 1 | 2.70 | 0.00 | 15.49 |  | 0 |  |  |  |  | 2 | 0.74 | 0.07 | 2.71 |
| Ankylosing spondylitis | 4 | **7.02** | **1.83** | **18.15** |  | 13 | **3.92** | **2.08** | **6.72** |  | 5 | 1.54 | 0.49 | 3.62 |  | 11 | **2.44** | **1.21** | **4.38** |  | 33 | **2.83** | **1.95** | **3.98** |
| Autoimmune hemolytic anemia | 1 | 6.67 | 0.00 | 38.22 |  | 2 | 3.17 | 0.30 | 11.67 |  | 0 |  |  |  |  | 1 | 2.94 | 0.00 | 16.86 |  | 4 | 2.56 | 0.67 | 6.63 |
| Behcet´s disease | 1 | 50.00 | 0.02 | 286.61 |  | 0 |  |  |  |  | 0 |  |  |  |  | 0 |  |  |  |  | 1 | 2.86 | 0.00 | 16.38 |
| Celiac disease | 1 | 3.85 | 0.00 | 22.05 |  | 3 | 2.50 | 0.47 | 7.40 |  | 0 |  |  |  |  | 6 | **5.13** | **1.85** | **11.24** |  | 10 | **2.67** | **1.27** | **4.94** |
| Chorea minor | 0 |  |  |  |  | 0 |  |  |  |  | 0 |  |  |  |  | 0 |  |  |  |  | 0 |  |  |  |
| Crohn´s disease | 4 | 2.15 | 0.56 | 5.56 |  | 20 | **2.02** | **1.23** | **3.13** |  | 9 | 1.00 | 0.45 | 1.91 |  | 13 | 1.55 | 0.82 | 2.66 |  | 46 | **1.58** | **1.16** | **2.11** |
| Diabetes mellitus type I | 0 |  |  |  |  | 1 | 2.13 | 0.00 | 12.20 |  | 1 | 2.08 | 0.00 | 11.94 |  | 1 | 1.20 | 0.00 | 6.91 |  | 3 | 1.62 | 0.31 | 4.80 |
| Discoid lupus erythematosus | 1 | 50.00 | 0.02 | 286.61 |  | 0 |  |  |  |  | 0 |  |  |  |  | 0 |  |  |  |  | 1 | 3.45 | 0.00 | 19.77 |
| Grave´s disease | 0 |  |  |  |  | 22 | **2.95** | **1.84** | **4.47** |  | 11 | 1.59 | 0.79 | 2.86 |  | 4 | 0.68 | 0.18 | 1.75 |  | 37 | **1.72** | **1.21** | **2.38** |
| Hashimoto´s thyroiditis | 2 | 2.70 | 0.25 | 9.94 |  | 5 | 1.59 | 0.50 | 3.73 |  | 4 | 2.11 | 0.55 | 5.44 |  | 3 | 1.80 | 0.34 | 5.32 |  | 14 | **1.88** | **1.02** | **3.16** |
| Immune thrombocytopenic purpura | 3 | **5.88** | **1.11** | **17.41** |  | 5 | 2.25 | 0.71 | 5.30 |  | 4 | 3.23 | 0.84 | 8.34 |  | 1 | 1.64 | 0.00 | 9.40 |  | 13 | **2.84** | **1.51** | **4.87** |
| Localized scleroderma | 0 |  |  |  |  | 0 |  |  |  |  | 0 |  |  |  |  | 1 | 5.56 | 0.00 | 31.85 |  | 1 | 1.85 | 0.00 | 10.62 |
| Lupoid hepatitis | 0 |  |  |  |  | 0 |  |  |  |  | 0 |  |  |  |  | 0 |  |  |  |  | 0 |  |  |  |
| Multiple sclerosis | 2 | 1.94 | 0.18 | 7.14 |  | 7 | 1.40 | 0.56 | 2.91 |  | 3 | 0.82 | 0.16 | 2.44 |  | 3 | 1.20 | 0.23 | 3.55 |  | 15 | 1.23 | 0.69 | 2.04 |
| Myasthenia gravis | 1 | 2.08 | 0.00 | 11.94 |  | 4 | 1.77 | 0.46 | 4.58 |  | 4 | 2.40 | 0.62 | 6.19 |  | 0 |  |  |  |  | 9 | 1.67 | 0.76 | 3.18 |
| Pernicious anemia | 2 | 2.15 | 0.20 | 7.91 |  | 6 | 1.11 | 0.40 | 2.43 |  | 7 | 1.56 | 0.62 | 3.22 |  | 4 | 1.04 | 0.27 | 2.68 |  | 19 | 1.29 | 0.78 | 2.02 |
| Polyarteritis nodosa | 0 |  |  |  |  | 0 |  |  |  |  | 1 | 1.18 | 0.00 | 6.74 |  | 0 |  |  |  |  | 1 | 0.37 | 0.00 | 2.13 |
| Polymyalgia rheumatica | 8 | 2.17 | 0.93 | 4.30 |  | 27 | 1.38 | 0.91 | 2.00 |  | 24 | **1.76** | **1.13** | **2.62** |  | 15 | 1.66 | 0.92 | 2.74 |  | 74 | **1.61** | **1.26** | **2.02** |
| Polymyositis/dermatomyositis | 1 | 5.88 | 0.00 | 33.72 |  | 1 | 1.45 | 0.00 | 8.31 |  | 1 | 2.17 | 0.00 | 12.46 |  | 0 |  |  |  |  | 3 | 1.92 | 0.36 | 5.69 |
| Primary biliary cirrhosis | 0 |  |  |  |  | 0 |  |  |  |  | 0 |  |  |  |  | 0 |  |  |  |  | 0 |  |  |  |
| Psoriasis | 6 | **3.45** | **1.24** | **7.56** |  | 17 | **1.76** | **1.02** | **2.82** |  | 12 | 1.44 | 0.74 | 2.52 |  | 14 | 1.58 | 0.86 | 2.66 |  | 49 | **1.71** | **1.27** | **2.26** |
| Reiter´s disease | 0 |  |  |  |  | 1 | 3.45 | 0.00 | 19.77 |  | 1 | 2.86 | 0.00 | 16.38 |  | 0 |  |  |  |  | 2 | 1.60 | 0.15 | 5.88 |
| Rheumatic fever | 1 | 16.67 | 0.01 | 95.54 |  | 0 |  |  |  |  | 0 |  |  |  |  | 1 | 2.86 | 0.00 | 16.38 |  | 2 | 1.96 | 0.18 | 7.21 |
| Rheumatoid arthritis | 18 | **2.80** | **1.66** | **4.44** |  | 56 | **1.83** | **1.38** | **2.37** |  | 34 | **1.85** | **1.28** | **2.59** |  | 15 | 1.34 | 0.75 | 2.21 |  | 123 | **1.85** | **1.53** | **2.20** |
| Sarcoidosis | 3 | 4.69 | 0.88 | 13.88 |  | 5 | 1.54 | 0.49 | 3.63 |  | 9 | **2.84** | **1.29** | **5.41** |  | 2 | 0.54 | 0.05 | 1.98 |  | 19 | **1.76** | **1.06** | **2.76** |
| Sjögren´s syndrome | 0 |  |  |  |  | 0 |  |  |  |  | 0 |  |  |  |  | 0 |  |  |  |  | 0 |  |  |  |
| Systemic lupus erythematosus | 3 | **9.38** | **1.77** | **27.75** |  | 3 | 2.14 | 0.40 | 6.34 |  | 1 | 0.94 | 0.00 | 5.41 |  | 2 | 2.25 | 0.21 | 8.26 |  | 9 | **2.45** | **1.11** | **4.68** |
| Systemic sclerosis | 0 |  |  |  |  | 2 | 3.51 | 0.33 | 12.90 |  | 2 | 6.25 | 0.59 | 22.99 |  | 1 | 4.55 | 0.00 | 26.06 |  | 5 | **3.85** | **1.21** | **9.05** |
| Ulcerative colitis | 4 | 1.36 | 0.35 | 3.52 |  | 20 | 1.17 | 0.71 | 1.81 |  | 16 | 1.08 | 0.61 | 1.75 |  | 20 | 1.37 | 0.83 | 2.12 |  | 60 | 1.21 | 0.92 | 1.56 |
| Wegener´s granulomatosis | 5 | **8.93** | **2.82** | **21.00** |  | 1 | 0.51 | 0.00 | 2.91 |  | 2 | 1.75 | 0.17 | 6.45 |  | 0 |  |  |  |  | 8 | 1.77 | 0.76 | 3.51 |
| All | 73 | **2.74** | **2.15** | **3.45** |  | 222 | **1.69** | **1.48** | **1.93** |  | 153 | **1.54** | **1.31** | **1.81** |  | 118 | **1.41** | **1.17** | **1.69** |  | 566 | **1.66** | **1.53** | **1.80** |
| O = observed number of cases; SIR = standardized incidence ratio; CI = confidence interval. | | | | | | | | | |  |  |  |  |  |  |  |  |  |  |  |  |  |  |  |
| Bold type: 95% CI does not include 1.00.  Adjusted for age, period, socioeconomic status, region of residence, hospitalization of chronic lower respiratory diseases, obesity, alcoholism, hypertension, diabetes atrial fibrillation, heart failure, renal disease, sepsis, and coronary heart disease. | | | | | | | | | | | | | | | | | | | | | | | | |

| **Supplement Table 3. SIR for subsequent hemorrhagic stroke of female patients with IMD** | | | | | | | | | | | | | | | | | | | | | | | | |
| --- | --- | --- | --- | --- | --- | --- | --- | --- | --- | --- | --- | --- | --- | --- | --- | --- | --- | --- | --- | --- | --- | --- | --- | --- |
|  | Follow-up interval (years) | | | | | | | | | | | | | | | | | | |  |  |  |  |  |
|  | <1 | | | |  | 1-5 | | | |  | 5-10 | | | |  | >=10 | | | |  | All | | | |
| Immune-mediated diseases | O | SIR | 95% CI | |  | O | SIR | 95% CI | |  | O | SIR | 95% CI | |  | O | SIR | 95% CI | |  | O | SIR | 95% CI | |
| Addison´s disease | 1 | 3.23 | 0.00 | 18.49 |  | 1 | 0.60 | 0.00 | 3.41 |  | 0 |  |  |  |  | 1 | 1.02 | 0.00 | 5.85 |  | 3 | 0.71 | 0.13 | 2.09 |
| Amyotrophic lateral sclerosis | 0 |  |  |  |  | 2 | 3.33 | 0.31 | 12.26 |  | 0 |  |  |  |  | 0 |  |  |  |  | 2 | 1.12 | 0.11 | 4.13 |
| Ankylosing spondylitis | 2 | **11.76** | **1.11** | **43.27** |  | 2 | 1.90 | 0.18 | 7.00 |  | 2 | 2.06 | 0.19 | 7.58 |  | 3 | 1.85 | 0.35 | 5.48 |  | 9 | **2.36** | **1.07** | **4.50** |
| Autoimmune hemolytic anemia | 0 |  |  |  |  | 2 | 2.78 | 0.26 | 10.22 |  | 1 | 2.33 | 0.00 | 13.33 |  | 1 | 3.03 | 0.00 | 17.37 |  | 4 | 2.42 | 0.63 | 6.27 |
| Behcet´s disease | 0 |  |  |  |  | 0 |  |  |  |  | 0 |  |  |  |  | 0 |  |  |  |  | 0 |  |  |  |
| Celiac disease | 1 | 3.33 | 0.00 | 19.11 |  | 3 | 1.79 | 0.34 | 5.29 |  | 7 | **4.70** | **1.86** | **9.73** |  | 2 | 1.08 | 0.10 | 3.98 |  | 13 | **2.44** | **1.30** | **4.19** |
| Chorea minor | 0 |  |  |  |  | 0 |  |  |  |  | 0 |  |  |  |  | 0 |  |  |  |  | 0 |  |  |  |
| Crohn´s disease | 1 | 0.65 | 0.00 | 3.72 |  | 27 | **3.30** | **2.17** | **4.80** |  | 10 | 1.44 | 0.69 | 2.66 |  | 11 | 1.59 | 0.79 | 2.86 |  | 49 | **2.08** | **1.54** | **2.75** |
| Diabetes mellitus type I | 0 |  |  |  |  | 1 | 3.03 | 0.00 | 17.37 |  | 0 |  |  |  |  | 1 | 0.86 | 0.00 | 4.94 |  | 2 | 1.03 | 0.10 | 3.77 |
| Discoid lupus erythematosus | 0 |  |  |  |  | 1 | 3.13 | 0.00 | 17.91 |  | 0 |  |  |  |  | 0 |  |  |  |  | 1 | 1.28 | 0.00 | 7.35 |
| Grave´s disease | 8 | 1.98 | 0.84 | 3.91 |  | 36 | **1.43** | **1.00** | **1.98** |  | 37 | **1.61** | **1.13** | **2.22** |  | 37 | **1.70** | **1.20** | **2.35** |  | 118 | **1.60** | **1.32** | **1.91** |
| Hashimoto´s thyroiditis | 2 | 1.01 | 0.09 | 3.70 |  | 22 | **2.14** | **1.34** | **3.25** |  | 15 | **2.10** | **1.17** | **3.47** |  | 8 | 1.26 | 0.54 | 2.49 |  | 47 | **1.82** | **1.34** | **2.43** |
| Immune thrombocytopenic purpura | 5 | **11.90** | **3.76** | **28.00** |  | 7 | **3.41** | **1.35** | **7.08** |  | 2 | 1.32 | 0.12 | 4.87 |  | 1 | 0.99 | 0.00 | 5.68 |  | 15 | **3.01** | **1.68** | **4.97** |
| Localized scleroderma | 0 |  |  |  |  | 1 | 1.11 | 0.00 | 6.37 |  | 1 | 0.99 | 0.00 | 5.68 |  | 5 | **4.13** | **1.30** | **9.72** |  | 7 | 2.15 | 0.85 | 4.45 |
| Lupoid hepatitis | 0 |  |  |  |  | 0 |  |  |  |  | 0 |  |  |  |  | 0 |  |  |  |  | 0 |  |  |  |
| Multiple sclerosis | 2 | 1.71 | 0.16 | 6.29 |  | 8 | 1.33 | 0.57 | 2.64 |  | 6 | 1.30 | 0.47 | 2.85 |  | 3 | 0.77 | 0.15 | 2.28 |  | 19 | 1.21 | 0.73 | 1.89 |
| Myasthenia gravis | 0 |  |  |  |  | 4 | 2.55 | 0.66 | 6.59 |  | 2 | 1.83 | 0.17 | 6.75 |  | 0 |  |  |  |  | 6 | 1.55 | 0.56 | 3.41 |
| Pernicious anemia | 2 | 2.15 | 0.20 | 7.91 |  | 12 | **2.24** | **1.15** | **3.92** |  | 6 | 1.49 | 0.54 | 3.26 |  | 3 | 0.94 | 0.18 | 2.78 |  | 23 | **1.70** | **1.08** | **2.56** |
| Polyarteritis nodosa | 2 | **12.50** | **1.18** | **45.97** |  | 0 |  |  |  |  | 2 | 2.78 | 0.26 | 10.22 |  | 0 |  |  |  |  | 4 | 1.72 | 0.45 | 4.44 |
| Polymyalgia rheumatica | 13 | **2.00** | **1.06** | **3.44** |  | 51 | **1.44** | **1.07** | **1.89** |  | 41 | **1.63** | **1.17** | **2.21** |  | 25 | 1.40 | 0.91 | 2.08 |  | 130 | **1.53** | **1.28** | **1.82** |
| Polymyositis/dermatomyositis | 0 |  |  |  |  | 2 | 2.35 | 0.22 | 8.65 |  | 0 |  |  |  |  | 1 | 4.17 | 0.00 | 23.88 |  | 3 | 1.78 | 0.33 | 5.25 |
| Primary biliary cirrhosis | 1 | 2.50 | 0.00 | 14.33 |  | 3 | 2.29 | 0.43 | 6.78 |  | 2 | 2.78 | 0.26 | 10.22 |  | 0 |  |  |  |  | 6 | 2.37 | 0.85 | 5.20 |
| Psoriasis | 3 | 2.17 | 0.41 | 6.44 |  | 15 | **1.93** | **1.07** | **3.18** |  | 11 | 1.59 | 0.79 | 2.86 |  | 7 | 0.99 | 0.39 | 2.05 |  | 36 | **1.56** | **1.09** | **2.16** |
| Reiter´s disease | 0 |  |  |  |  | 0 |  |  |  |  | 0 |  |  |  |  | 0 |  |  |  |  | 0 |  |  |  |
| Rheumatic fever | 0 |  |  |  |  | 0 |  |  |  |  | 0 |  |  |  |  | 0 |  |  |  |  | 0 |  |  |  |
| Rheumatoid arthritis | 47 | **3.49** | **2.57** | **4.65** |  | 135 | **2.14** | **1.79** | **2.53** |  | 75 | **1.95** | **1.53** | **2.44** |  | 46 | **2.00** | **1.46** | **2.67** |  | 303 | **2.19** | **1.95** | **2.45** |
| Sarcoidosis | 0 |  |  |  |  | 7 | 2.21 | 0.88 | 4.58 |  | 5 | 1.66 | 0.52 | 3.89 |  | 4 | 1.03 | 0.27 | 2.67 |  | 16 | 1.51 | 0.86 | 2.45 |
| Sjögren´s syndrome | 0 |  |  |  |  | 3 | 1.55 | 0.29 | 4.58 |  | 2 | 1.17 | 0.11 | 4.30 |  | 0 |  |  |  |  | 5 | 0.92 | 0.29 | 2.15 |
| Systemic lupus erythematosus | 6 | **8.33** | **3.00** | **18.26** |  | 10 | **3.23** | **1.54** | **5.96** |  | 3 | 1.28 | 0.24 | 3.78 |  | 4 | 1.78 | 0.46 | 4.60 |  | 23 | **2.73** | **1.73** | **4.10** |
| Systemic sclerosis | 2 | 4.55 | 0.43 | 16.72 |  | 3 | 2.31 | 0.44 | 6.83 |  | 1 | 1.28 | 0.00 | 7.35 |  | 1 | 2.78 | 0.00 | 15.92 |  | 7 | 2.43 | 0.96 | 5.04 |
| Ulcerative colitis | 3 | 1.59 | 0.30 | 4.70 |  | 20 | **1.89** | **1.15** | **2.93** |  | 12 | 1.43 | 0.74 | 2.51 |  | 13 | 1.56 | 0.83 | 2.67 |  | 48 | **1.65** | **1.21** | **2.18** |
| Wegener´s granulomatosis | 1 | 2.13 | 0.00 | 12.20 |  | 2 | 1.48 | 0.14 | 5.45 |  | 0 |  |  |  |  | 0 |  |  |  |  | 3 | 1.00 | 0.19 | 2.97 |
| All | 102 | **2.60** | **2.12** | **3.15** |  | 380 | **1.92** | **1.73** | **2.13** |  | 243 | **1.69** | **1.48** | **1.91** |  | 177 | **1.51** | **1.30** | **1.75** |  | 902 | **1.81** | **1.69** | **1.93** |
| O = observed number of cases; SIR = standardized incidence ratio; CI = confidence interval. | | | | | | | | | |  |  |  |  |  |  |  |  |  |  |  |  |  |  |  |
| Bold type: 95% CI does not include 1.00.  Adjusted for age, period, socioeconomic status, region of residence, hospitalization of chronic lower respiratory diseases, obesity, alcoholism, hypertension, diabetes, atrial fibrillation, heart failure, renal disease, sepsis, and coronary heart disease. | | | | | | | | | | | | | | | | | | | | | | | | |

| **Table 4. SIR for subsequent hemorrhagic stroke of patients with IMD after one year of follow-up** | | | | | | | | | | | | | | | | | | | |
| --- | --- | --- | --- | --- | --- | --- | --- | --- | --- | --- | --- | --- | --- | --- | --- | --- | --- | --- | --- |
|  | Age at diagnosis of hemorrhagic stroke (years) | | | | | | | | | | | | | | | | | | |
|  | <50 | | | |  | 50-59 | | | |  | 60-69 | | | |  | >=70 | | | |
| Immune-mediated diseases | O | SIR | 95% CI | |  | O | SIR | 95% CI | |  | O | SIR | 95% CI | |  | O | SIR | 95% CI | |
| Addison´s disease | 1 | 2.04 | 0.00 | 11.70 |  | 1 | 1.27 | 0.00 | 7.26 |  | 0 |  |  |  |  | 2 | 0.42 | 0.04 | 1.56 |
| Amyotrophic lateral sclerosis | 0 |  |  |  |  | 0 |  |  |  |  | 0 |  |  |  |  | 3 | 1.67 | 0.31 | 4.93 |
| Ankylosing spondylitis | 4 | 2.96 | 0.77 | 7.66 |  | 11 | **3.63** | **1.80** | **6.52** |  | 9 | 1.75 | 0.80 | 3.34 |  | 12 | **2.31** | **1.19** | **4.04** |
| Autoimmune hemolytic anemia | 2 | **20.00** | **1.89** | **73.55** |  | 0 |  |  |  |  | 0 |  |  |  |  | 5 | 2.16 | 0.68 | 5.07 |
| Behcet´s disease | 0 |  |  |  |  | 0 |  |  |  |  | 0 |  |  |  |  | 0 |  |  |  |
| Celiac disease | 1 | 0.78 | 0.00 | 4.44 |  | 0 |  |  |  |  | 5 | **3.21** | **1.01** | **7.54** |  | 15 | **3.05** | **1.70** | **5.05** |
| Chorea minor | 0 |  |  |  |  | 0 |  |  |  |  | 0 |  |  |  |  | 0 |  |  |  |
| Crohn disease | 11 | **2.24** | **1.11** | **4.02** |  | 20 | 2.47 | 1.51 | 3.82 |  | 14 | 1.11 | 0.60 | 1.86 |  | 45 | **1.91** | **1.39** | **2.55** |
| Diabetes mellitus type I | 5 | 1.36 | 0.43 | 3.19 |  | 0 |  |  |  |  | 0 |  |  |  |  | 0 |  |  |  |
| Discoid lupus erythematosus | 0 |  |  |  |  | 0 |  |  |  |  | 0 |  |  |  |  | 1 | 1.72 | 0.00 | 9.88 |
| Grave´s disease | 6 | 1.30 | 0.47 | 2.84 |  | 11 | 1.52 | 0.75 | 2.72 |  | 26 | **1.95** | **1.27** | **2.86** |  | 104 | **1.60** | **1.31** | **1.94** |
| Hashimoto´s thyroiditis | 2 | 3.45 | 0.33 | 12.68 |  | 2 | 1.11 | 0.10 | 4.09 |  | 6 | 1.55 | 0.56 | 3.40 |  | 47 | **1.94** | **1.43** | **2.58** |
| Immune thrombocytopenic purpura | 2 | 3.77 | 0.36 | 13.88 |  | 2 | 3.85 | 0.36 | 14.14 |  | 3 | 2.08 | 0.39 | 6.17 |  | 13 | **2.11** | **1.12** | **3.63** |
| Localized scleroderma | 0 |  |  |  |  | 0 |  |  |  |  | 0 |  |  |  |  | 8 | **2.62** | **1.12** | **5.19** |
| Lupoid hepatitis | 0 |  |  |  |  | 0 |  |  |  |  | 0 |  |  |  |  | 0 |  |  |  |
| Multiple sclerosis | 4 | 2.60 | 0.68 | 6.72 |  | 5 | 1.14 | 0.36 | 2.68 |  | 12 | 1.68 | 0.86 | 2.94 |  | 9 | 0.72 | 0.33 | 1.37 |
| Myasthenia gravis | 1 | 4.55 | 0.00 | 26.06 |  | 1 | 2.13 | 0.00 | 12.20 |  | 3 | 2.27 | 0.43 | 6.73 |  | 9 | 1.40 | 0.63 | 2.67 |
| Pernicious anemia | 0 |  |  |  |  | 1 | 2.33 | 0.00 | 13.33 |  | 2 | 1.04 | 0.10 | 3.83 |  | 35 | **1.47** | **1.02** | **2.04** |
| Polyarteritis nodosa | 1 | 4.76 | 0.00 | 27.30 |  | 0 |  |  |  |  | 0 |  |  |  |  | 2 | 0.64 | 0.06 | 2.35 |
| Polymyalgia rheumatica | 0 |  |  |  |  | 1 | 1.11 | 0.00 | 6.37 |  | 7 | 0.97 | 0.39 | 2.02 |  | 175 | **1.55** | **1.33** | **1.80** |
| Polymyositis/dermatomyositis | 0 |  |  |  |  | 1 | 4.00 | 0.00 | 22.93 |  | 0 |  |  |  |  | 4 | 2.02 | 0.53 | 5.22 |
| Primary biliary cirrhosis | 0 |  |  |  |  | 3 | **8.82** | **1.66** | **26.12** |  | 16 | **1.85** | **1.06** | **3.01** |  | 2 | 1.47 | 0.14 | 5.41 |
| Psoriasis | 0 |  |  |  |  | 14 | **2.18** | **1.19** | **3.66** |  | 12 | **3.00** | **1.54** | **5.26** |  | 36 | 1.27 | 0.89 | 1.76 |
| Reiter´s disease | 0 |  |  |  |  | 0 |  |  |  |  | 0 |  |  |  |  | 0 |  |  |  |
| Rheumatic fever | 0 |  |  |  |  | 0 |  |  |  |  | 25 | **1.99** | **1.29** | **2.94** |  | 1 | 0.82 | 0.00 | 4.70 |
| Rheumatoid arthritis | 6 | 2.64 | 0.95 | 5.79 |  | 10 | 1.15 | 0.55 | 2.13 |  | 40 | **2.29** | **1.64** | **3.12** |  | 285 | **1.94** | **1.72** | **2.18** |
| Sarcoidosis | 2 | 1.01 | 0.10 | 3.71 |  | 5 | 1.73 | 0.55 | 4.07 |  | 5 | 2.56 | 0.81 | 6.03 |  | 15 | 1.38 | 0.77 | 2.29 |
| Sjögren´s syndrome | 0 |  |  |  |  | 0 |  |  |  |  | 2 | 1.12 | 0.11 | 4.13 |  | 5 | 1.15 | 0.36 | 2.70 |
| Systemic lupus erythematosus | 6 | **7.50** | **2.70** | **16.43** |  | 4 | 3.10 | 0.81 | 8.02 |  | 2 | 1.05 | 0.10 | 3.85 |  | 10 | 1.57 | 0.75 | 2.91 |
| Systemic sclerosis | 1 | 10.00 | 0.00 | 57.32 |  | 0 |  |  |  |  | 18 | 1.47 | 0.87 | 2.32 |  | 6 | 2.43 | 0.87 | 5.32 |
| Ulcerative colitis | 6 | 0.82 | 0.30 | 1.80 |  | 9 | 0.83 | 0.38 | 1.59 |  | 8 | 1.61 | 0.69 | 3.19 |  | 63 | **1.59** | **1.22** | **2.03** |
| Wegener´s granulomatosis | 0 |  |  |  |  | 1 | 1.61 | 0.00 | 9.25 |  | 0 |  |  |  |  | 3 | 0.68 | 0.13 | 2.01 |
| All | 61 | **1.71** | **1.31** | **2.20** |  | 102 | **1.64** | **1.34** | **2.00** |  | 215 | **1.70** | **1.48** | **1.94** |  | 915 | **1.67** | **1.56** | **1.78** |
| O = observed number of cases; SIR = standardized incidence ratio; CI = confidence interval. | | | | | | | | | |  |  |  |  |  |  |  |  |  |  |
| Bold type: 95% CI does not include 1.00.  Adjusted for age, period, socioeconomic status, region of residence, hospitalization of chronic lower respiratory diseases, obesity, alcoholism, hypertension, diabetes, atrial fibrillation, heart failure, renal disease, sepsis, and coronary heart disease. | | | | | | | | | | | | | | | | | | | |

| **Supplement Table 5. SIR for subsequent hemorrhagic stroke of male patients with IMD after one year of follow-up** | | | | | | | | | | | | | | | | | |  |  |
| --- | --- | --- | --- | --- | --- | --- | --- | --- | --- | --- | --- | --- | --- | --- | --- | --- | --- | --- | --- |
|  | Age at diagnosis of hemorrhagic stroke (years) | | | | | | | | | | | | | | | | | | |
|  | <50 | | | |  | 50-59 | | | |  | 60-69 | | | |  | >=70 | | | |
| Immune-mediated diseases | O | SIR | 95% CI | |  | O | SIR | 95% CI | |  | O | SIR | 95% CI | |  | O | SIR | 95% CI | |
| Addison´s disease | 0 |  |  |  |  | 1 | 2.94 | 0.00 | 16.86 |  | 0 |  |  |  |  | 1 | 0.49 | 0.00 | 2.81 |
| Amyotrophic lateral sclerosis | 0 |  |  |  |  | 0 |  |  |  |  | 0 |  |  |  |  | 1 | 0.94 | 0.00 | 5.41 |
| Ankylosing spondylitis | 4 | 3.57 | 0.93 | 9.23 |  | 9 | **3.64** | **1.65** | **6.95** |  | 7 | 1.83 | 0.72 | 3.79 |  | 9 | **2.47** | **1.12** | **4.70** |
| Autoimmune hemolytic anemia | 1 | 25.00 | 0.01 | 143.31 |  | 0 |  |  |  |  | 0 |  |  |  |  | 2 | 1.87 | 0.18 | 6.87 |
| Behcet´s disease | 0 |  |  |  |  | 0 |  |  |  |  | 0 |  |  |  |  | 0 |  |  |  |
| Celiac disease | 1 | 1.61 | 0.00 | 9.25 |  | 0 |  |  |  |  | 4 | **4.76** | **1.24** | **12.31** |  | 4 | 2.41 | 0.63 | 6.23 |
| Chorea minor | 0 |  |  |  |  | 0 |  |  |  |  | 0 |  |  |  |  | 0 |  |  |  |
| Crohn´s disease | 3 | 1.02 | 0.19 | 3.01 |  | 10 | 1.98 | 0.94 | 3.65 |  | 10 | 1.27 | 0.61 | 2.35 |  | 19 | **1.67** | **1.00** | **2.61** |
| Diabetes mellitus type I | 3 | 1.69 | 0.32 | 4.99 |  | 0 |  |  |  |  | 0 |  |  |  |  | 0 |  |  |  |
| Discoid lupus erythematosus | 0 |  |  |  |  | 0 |  |  |  |  | 0 |  |  |  |  | 0 |  |  |  |
| Grave´s disease | 2 | 2.00 | 0.19 | 7.36 |  | 5 | 2.69 | 0.85 | 6.32 |  | 9 | 2.16 | 0.98 | 4.12 |  | 21 | 1.58 | 0.98 | 2.42 |
| Hashimoto´s thyroiditis | 0 |  |  |  |  | 0 |  |  |  |  | 0 | 0.00 | 0.79 | 3.24 |  | 12 | **2.51** | **1.29** | **4.39** |
| Immune thrombocytopenic purpura | 1 | 3.70 | 0.00 | 21.23 |  | 1 | 3.33 | 0.00 | 19.11 |  | 2 | 2.33 | 0.22 | 8.55 |  | 6 | 2.28 | 0.82 | 5.00 |
| Localized scleroderma | 0 |  |  |  |  | 0 |  |  |  |  | 0 |  |  |  |  | 1 | 3.03 | 0.00 | 17.37 |
| Lupoid hepatitis | 0 |  |  |  |  | 0 |  |  |  |  | 0 |  |  |  |  | 0 |  |  |  |
| Multiple sclerosis | 3 | 4.35 | 0.82 | 12.87 |  | 3 | 1.41 | 0.27 | 4.17 |  | 4 | 1.13 | 0.29 | 2.93 |  | 3 | 0.63 | 0.12 | 1.87 |
| Myasthenia gravis | 0 |  |  |  |  | 0 |  |  |  |  | 2 | 2.33 | 0.22 | 8.55 |  | 6 | 1.60 | 0.57 | 3.50 |
| Pernicious anemia | 0 |  |  |  |  | 0 |  |  |  |  | 1 | 0.75 | 0.00 | 4.28 |  | 16 | 1.33 | 0.76 | 2.16 |
| Polyarteritis nodosa | 1 | 25.00 | 0.01 | 143.31 |  | 0 |  |  |  |  | 0 |  |  |  |  | 0 |  |  |  |
| Polymyalgia rheumatica | 0 |  |  |  |  | 1 | 1.82 | 0.00 | 10.42 |  | 5 | 1.31 | 0.41 | 3.08 |  | 60 | **1.58** | **1.21** | **2.04** |
| Polymyositis/dermatomyositis | 0 |  |  |  |  | 0 |  |  |  |  | 0 |  |  |  |  | 2 | 2.33 | 0.22 | 8.55 |
| Primary biliary cirrhosis | 0 |  |  |  |  | 0 |  |  |  |  | 16 | **2.02** | **1.15** | **3.28** |  | 0 |  |  |  |
| Psoriasis | 0 |  |  |  |  | 8 | 1.82 | 0.78 | 3.60 |  | 2 | 6.06 | 0.57 | 22.29 |  | 19 | 1.46 | 0.88 | 2.29 |
| Reiter´s disease | 0 |  |  |  |  | 0 |  |  |  |  | 0 |  |  |  |  | 0 |  |  |  |
| Rheumatic fever | 0 |  |  |  |  | 0 |  |  |  |  | 25 | **2.00** | **1.29** | **2.96** |  | 1 | 1.79 | 0.00 | 10.24 |
| Rheumatoid arthritis | 5 | **5.26** | **1.66** | **12.38** |  | 4 | 1.00 | 0.26 | 2.59 |  | 5 | 1.89 | 0.60 | 4.46 |  | 71 | **1.66** | **1.30** | **2.09** |
| Sarcoidosis | 2 | 1.32 | 0.12 | 4.84 |  | 3 | 1.46 | 0.27 | 4.31 |  | 0 |  |  |  |  | 6 | 1.53 | 0.55 | 3.36 |
| Sjögren´s syndrome | 0 |  |  |  |  | 0 |  |  |  |  | 2 | 2.02 | 0.19 | 7.43 |  | 0 |  |  |  |
| Systemic lupus erythematosus | 1 | 3.70 | 0.00 | 21.23 |  | 0 |  |  |  |  | 1 | 3.57 | 0.00 | 20.47 |  | 3 | 1.69 | 0.32 | 5.02 |
| Systemic sclerosis | 1 | 25.00 | 0.01 | 143.31 |  | 0 |  |  |  |  | 16 | 1.35 | 0.77 | 2.19 |  | 3 | 4.48 | 0.84 | 13.25 |
| Ulcerative colitis | 4 | 0.79 | 0.20 | 2.04 |  | 5 | 0.66 | 0.21 | 1.55 |  | 1 | 1.15 | 0.00 | 6.59 |  | 31 | 1.41 | 0.96 | 2.00 |
| Wegener´s granulomatosis | 0 |  |  |  |  | 1 | 2.04 | 0.00 | 11.70 |  | 0 |  |  |  |  | 1 | 0.40 | 0.00 | 2.30 |
| All | 32 | **1.66** | **1.13** | **2.34** |  | 51 | **1.48** | **1.10** | **1.95** |  | 112 | **1.63** | **1.34** | **1.96** |  | 298 | **1.56** | **1.39** | **1.74** |
| O = observed number of cases; SIR = standardized incidence ratio; CI = confidence interval. | | | | | | | | | |  |  |  |  |  |  |  |  |  |  |
| Bold type: 95% CI does not include 1.00.  Adjusted for age, period, socioeconomic status, region of residence, hospitalization of chronic lower respiratory diseases, obesity, alcoholism, hypertension, diabetes, atrial fibrillation, heart failure, renal disease, sepsis, and coronary heart disease | | | | | | | | | | | | | | | | | | | |

| **Supplement Table 6. SIR for subsequent hemorrhagic stroke of female patients with IMD after one year of follow-up** | | | | | | | | | | | | | | | | | |  |  |
| --- | --- | --- | --- | --- | --- | --- | --- | --- | --- | --- | --- | --- | --- | --- | --- | --- | --- | --- | --- |
|  | Age at diagnosis of hemorrhagic stroke (years) | | | | | | | | | | | | | | | | | | |
|  | <50 | | | |  | 50-59 | | | |  | 60-69 | | | |  | >=70 | | | |
| Immune-mediated diseases | O | SIR | 95% CI | |  | O | SIR | 95% CI | |  | O | SIR | 95% CI | |  | O | SIR | 95% CI | |
| Addison´s disease | 1 | 5.26 | 0.00 | 30.17 |  | 0 |  |  |  |  | 0 |  |  |  |  | 1 | 0.37 | 0.00 | 2.15 |
| Amyotrophic lateral sclerosis | 0 |  |  |  |  | 0 |  |  |  |  | 0 |  |  |  |  | 2 | 2.70 | 0.25 | 9.94 |
| Ankylosing spondylitis | 0 |  |  |  |  | 2 | 3.57 | 0.34 | 13.13 |  | 2 | 1.54 | 0.15 | 5.66 |  | 3 | 1.94 | 0.36 | 5.73 |
| Autoimmune hemolytic anemia | 1 | 16.67 | 0.01 | 95.54 |  | 0 |  |  |  |  | 0 |  |  |  |  | 3 | 2.40 | 0.45 | 7.10 |
| Behcet´s disease | 0 |  |  |  |  | 0 |  |  |  |  | 0 |  |  |  |  | 0 |  |  |  |
| Celiac disease | 0 | 0.00 | 1.43 | 5.85 |  | 0 |  |  |  |  | 1 | 1.39 | 0.00 | 7.96 |  | 11 | **3.38** | **1.68** | **6.08** |
| Chorea minor | 0 |  |  |  |  | 0 |  |  |  |  | 0 |  |  |  |  | 0 |  |  |  |
| Crohn´s disease | 8 | **4.08** | **1.74** | **8.08** |  | 10 | **3.29** | **1.57** | **6.07** |  | 4 | 0.84 | 0.22 | 2.16 |  | 26 | **2.12** | **1.39** | **3.12** |
| Diabetes mellitus type I | 2 | 1.05 | 0.10 | 3.85 |  | 0 |  |  |  |  | 0 |  |  |  |  | 0 |  |  |  |
| Discoid lupus erythematosus | 0 |  |  |  |  | 0 |  |  |  |  | 0 |  |  |  |  | 1 | 2.22 | 0.00 | 12.74 |
| Grave´s disease | 4 | 1.10 | 0.29 | 2.85 |  | 6 | 1.11 | 0.40 | 2.43 |  | 17 | **1.85** | **1.07** | **2.96** |  | 83 | **1.61** | **1.28** | **1.99** |
| Hashimoto´s thyroiditis | 2 | 4.88 | 0.46 | 17.94 |  | 2 | 1.59 | 0.15 | 5.84 |  | 6 | 2.26 | 0.81 | 4.94 |  | 35 | **1.80** | **1.25** | **2.51** |
| Immune thrombocytopenic purpura | 1 | 3.85 | 0.00 | 22.05 |  | 1 | 4.55 | 0.00 | 26.06 |  | 1 | 1.72 | 0.00 | 9.88 |  | 7 | 1.99 | 0.79 | 4.12 |
| Localized scleroderma | 0 |  |  |  |  | 0 |  |  |  |  | 0 |  |  |  |  | 7 | **2.57** | **1.02** | **5.33** |
| Lupoid hepatitis | 0 |  |  |  |  | 0 |  |  |  |  | 0 |  |  |  |  | 0 |  |  |  |
| Multiple sclerosis | 1 | 1.18 | 0.00 | 6.74 |  | 2 | 0.88 | 0.08 | 3.25 |  | 8 | 2.21 | 0.94 | 4.38 |  | 6 | 0.77 | 0.28 | 1.69 |
| Myasthenia gravis | 1 | 7.14 | 0.00 | 40.94 |  | 1 | 4.17 | 0.00 | 23.88 |  | 1 | 2.17 | 0.00 | 12.46 |  | 3 | 1.12 | 0.21 | 3.33 |
| Pernicious anemia | 0 |  |  |  |  | 1 | 6.67 | 0.00 | 38.22 |  | 1 | 1.72 | 0.00 | 9.88 |  | 19 | 1.61 | 0.97 | 2.52 |
| Polyarteritis nodosa | 0 |  |  |  |  | 0 |  |  |  |  | 0 |  |  |  |  | 2 | 1.23 | 0.12 | 4.54 |
| Polymyalgia rheumatica | 0 |  |  |  |  | 0 |  |  |  |  | 2 | 0.60 | 0.06 | 2.19 |  | 115 | **1.54** | **1.27** | **1.85** |
| Polymyositis/dermatomyositis | 0 |  |  |  |  | 1 | 9.09 | 0.00 | 52.11 |  | 0 |  |  |  |  | 2 | 1.79 | 0.17 | 6.57 |
| Primary biliary cirrhosis | 0 |  |  |  |  | 3 | **12.50** | **2.36** | **37.00** |  | 0 |  |  |  |  | 2 | 1.74 | 0.16 | 6.40 |
| Psoriasis | 0 |  |  |  |  | 6 | **2.96** | **1.06** | **6.48** |  | 10 | **2.72** | **1.30** | **5.03** |  | 17 | 1.11 | 0.64 | 1.78 |
| Reiter´s disease | 0 |  |  |  |  | 0 |  |  |  |  | 0 |  |  |  |  | 0 |  |  |  |
| Rheumatic fever | 0 |  |  |  |  | 0 |  |  |  |  | 0 |  |  |  |  | 0 |  |  |  |
| Rheumatoid arthritis | 1 | 0.76 | 0.00 | 4.34 |  | 6 | 1.28 | 0.46 | 2.82 |  | 35 | **2.36** | **1.65** | **3.29** |  | 214 | **2.06** | **1.79** | **2.35** |
| Sarcoidosis | 0 |  |  |  |  | 2 | 2.41 | 0.23 | 8.86 |  | 5 | 2.73 | 0.86 | 6.43 |  | 9 | 1.30 | 0.59 | 2.47 |
| Sjögren´s syndrome | 0 |  |  |  |  | 0 |  |  |  |  | 0 |  |  |  |  | 5 | 1.29 | 0.41 | 3.04 |
| Systemic lupus erythematosus | 5 | **9.43** | **2.98** | **22.19** |  | 4 | **4.17** | **1.08** | **10.77** |  | 1 | 0.61 | 0.00 | 3.52 |  | 7 | 1.53 | 0.61 | 3.17 |
| Systemic sclerosis | 0 |  |  |  |  | 0 |  |  |  |  | 2 | 5.13 | 0.48 | 18.86 |  | 3 | 1.67 | 0.31 | 4.93 |
| Ulcerative colitis | 2 | 0.89 | 0.08 | 3.28 |  | 4 | 1.24 | 0.32 | 3.20 |  | 7 | 1.71 | 0.68 | 3.54 |  | 32 | **1.81** | **1.24** | **2.55** |
| Wegener´s granulomatosis | 0 |  |  |  |  | 0 |  |  |  |  | 0 |  |  |  |  | 2 | 1.04 | 0.10 | 3.83 |
| All | 29 | **1.78** | **1.19** | **2.56** |  | 51 | **1.85** | **1.38** | **2.43** |  | 103 | **1.79** | **1.46** | **2.17** |  | 617 | **1.73** | **1.59** | **1.87** |
| O = observed number of cases; SIR = standardized incidence ratio; CI = confidence interval. | | | | | | | | | |  |  |  |  |  |  |  |  |  |  |
| Bold type: 95% CI does not include 1.00.  Adjusted for age, period, socioeconomic status, region of residence, hospitalization of chronic lower respiratory diseases, obesity, alcoholism, hypertension, diabetes, atrial fibrillation, heart failure, renal disease, sepsis, and coronary heart disease. | | | | | | | | | | | | | | | | | | | |

| **Supplement Table 7. SIR for subsequent ischemic stroke of male patients with IMD** | | | | | | | | | | | | | | | | | | | | | | | | |
| --- | --- | --- | --- | --- | --- | --- | --- | --- | --- | --- | --- | --- | --- | --- | --- | --- | --- | --- | --- | --- | --- | --- | --- | --- |
|  | Follow-up interval (years) | | | | | | | | | | | | | | | | | | |  |  |  |  |  |
|  | <1 | | | |  | 1-5 | | | |  | 5-10 | | | |  | >=10 | | | |  | All | | | |
| Immune-mediated diseases | O | SIR | 95% CI | |  | O | SIR | 95% CI | |  | O | SIR | 95% CI | |  | O | SIR | 95% CI | |  | O | SIR | 95% CI | |
| Addison´s disease | 6 | **2.88** | **1.04** | **6.32** |  | 11 | 1.07 | 0.53 | 1.91 |  | 9 | 1.35 | 0.61 | 2.58 |  | 4 | 0.88 | 0.23 | 2.28 |  | 30 | 1.27 | 0.86 | 1.82 |
| Amyotrophic lateral sclerosis | 6 | 0.84 | 0.30 | 1.84 |  | 11 | 1.78 | 0.88 | 3.20 |  | 6 | 2.40 | 0.86 | 5.26 |  | 2 | 1.75 | 0.17 | 6.45 |  | 25 | 1.47 | 0.95 | 2.18 |
| Ankylosing spondylitis | 5 | 1.40 | 0.44 | 3.30 |  | 33 | **1.60** | **1.10** | **2.25** |  | 15 | 0.83 | 0.46 | 1.37 |  | 25 | 1.06 | 0.68 | 1.56 |  | 78 | 1.18 | 0.94 | 1.48 |
| Autoimmune hemolytic anemia | 1 | 0.87 | 0.00 | 4.98 |  | 5 | 0.95 | 0.30 | 2.23 |  | 11 | **3.01** | **1.49** | **5.40** |  | 4 | 2.02 | 0.53 | 5.22 |  | 21 | **1.74** | **1.08** | **2.66** |
| Behcet´s disease | 0 |  |  |  |  | 0 |  |  |  |  | 0 |  |  |  |  | 0 |  |  |  |  | 0 |  |  |  |
| Celiac disease | 3 | 1.65 | 0.31 | 4.88 |  | 14 | 1.67 | 0.91 | 2.81 |  | 12 | 1.88 | 0.97 | 3.30 |  | 10 | 1.66 | 0.79 | 3.07 |  | 39 | **1.73** | **1.23** | **2.36** |
| Chorea minor | 0 |  |  |  |  | 0 |  |  |  |  | 0 |  |  |  |  | 0 |  |  |  |  | 0 |  |  |  |
| Crohn´s disease | 26 | **2.29** | **1.50** | **3.37** |  | 69 | 1.13 | 0.88 | 1.44 |  | 46 | 1.03 | 0.75 | 1.37 |  | 46 | 1.12 | 0.82 | 1.49 |  | 187 | **1.18** | **1.02** | **1.37** |
| Diabetes mellitus type I | 1 | 11.11 | 0.00 | 63.69 |  | 2 | 0.53 | 0.05 | 1.94 |  | 3 | 2.54 | 0.48 | 7.53 |  | 10 | **6.67** | **3.17** | **12.31** |  | 16 | **2.44** | **1.39** | **3.97** |
| Discoid lupus erythematosus | 1 | 6.25 | 0.00 | 35.83 |  | 1 | 1.28 | 0.00 | 7.35 |  | 0 |  |  |  |  | 1 | 1.92 | 0.00 | 11.02 |  | 3 | 1.45 | 0.27 | 4.29 |
| Grave´s disease | 13 | 1.38 | 0.73 | 2.36 |  | 89 | **1.53** | **1.23** | **1.89** |  | 56 | 1.18 | 0.89 | 1.53 |  | 49 | 1.32 | 0.98 | 1.75 |  | 207 | **1.36** | **1.18** | **1.56** |
| Hashimoto´s thyroiditis | 18 | **3.02** | **1.79** | **4.78** |  | 45 | **1.76** | **1.28** | **2.35** |  | 17 | 1.08 | 0.63 | 1.73 |  | 14 | 1.23 | 0.67 | 2.07 |  | 94 | **1.60** | **1.29** | **1.96** |
| Immune thrombocytopenic purpura | 7 | 2.11 | 0.84 | 4.37 |  | 32 | **2.27** | **1.55** | **3.21** |  | 9 | 1.15 | 0.52 | 2.19 |  | 6 | 1.72 | 0.62 | 3.78 |  | 54 | **1.88** | **1.41** | **2.46** |
| Localized scleroderma | 0 |  |  |  |  | 2 | 1.53 | 0.14 | 5.61 |  | 1 | 0.98 | 0.00 | 5.62 |  | 2 | 2.35 | 0.22 | 8.65 |  | 5 | 1.46 | 0.46 | 3.44 |
| Lupoid hepatitis | 1 | 7.14 | 0.00 | 40.94 |  | 3 | 5.17 | 0.98 | 15.31 |  | 0 |  |  |  |  | 0 |  |  |  |  | 4 | **4.40** | **1.14** | **11.37** |
| Multiple sclerosis | 15 | **2.54** | **1.42** | **4.20** |  | 30 | 1.00 | 0.68 | 1.43 |  | 21 | 1.05 | 0.65 | 1.61 |  | 14 | 1.07 | 0.58 | 1.80 |  | 80 | 1.16 | 0.92 | 1.45 |
| Myasthenia gravis | 4 | 1.24 | 0.32 | 3.21 |  | 18 | 1.15 | 0.68 | 1.82 |  | 13 | 1.18 | 0.63 | 2.03 |  | 6 | 0.97 | 0.35 | 2.13 |  | 41 | 1.14 | 0.82 | 1.55 |
| Pernicious anemia | 10 | 1.32 | 0.63 | 2.44 |  | 68 | **1.55** | **1.20** | **1.97** |  | 39 | 1.11 | 0.79 | 1.52 |  | 39 | **1.53** | **1.09** | **2.09** |  | 156 | **1.39** | **1.18** | **1.63** |
| Polyarteritis nodosa | 2 | 1.20 | 0.11 | 4.40 |  | 10 | 1.37 | 0.65 | 2.54 |  | 5 | 0.94 | 0.30 | 2.20 |  | 7 | 1.51 | 0.60 | 3.13 |  | 24 | 1.27 | 0.81 | 1.89 |
| Polymyalgia rheumatica | 45 | **1.42** | **1.03** | **1.90** |  | 246 | **1.47** | **1.29** | **1.67** |  | 183 | **1.74** | **1.50** | **2.01** |  | 94 | **1.57** | **1.27** | **1.92** |  | 568 | **1.56** | **1.44** | **1.70** |
| Polymyositis/dermatomyositis | 1 | 0.93 | 0.00 | 5.31 |  | 4 | 0.93 | 0.24 | 2.40 |  | 2 | 0.81 | 0.08 | 2.99 |  | 2 | 1.67 | 0.16 | 6.13 |  | 9 | 0.99 | 0.45 | 1.90 |
| Primary biliary cirrhosis | 1 | 1.72 | 0.00 | 9.88 |  | 2 | 1.46 | 0.14 | 5.37 |  | 1 | 1.32 | 0.00 | 7.54 |  | 0 |  |  |  |  | 4 | 1.43 | 0.37 | 3.71 |
| Psoriasis | 23 | **2.02** | **1.28** | **3.03** |  | 100 | **1.58** | **1.29** | **1.92** |  | 79 | **1.59** | **1.26** | **1.98** |  | 70 | **1.42** | **1.11** | **1.80** |  | 272 | **1.57** | **1.39** | **1.76** |
| Reiter´s disease | 0 |  |  |  |  | 3 | 1.60 | 0.30 | 4.75 |  | 5 | 2.76 | 0.87 | 6.50 |  | 2 | 0.62 | 0.06 | 2.29 |  | 10 | 1.39 | 0.66 | 2.57 |
| Rheumatic fever | 2 | 3.39 | 0.32 | 12.47 |  | 3 | 1.12 | 0.21 | 3.30 |  | 6 | 2.58 | 0.93 | 5.64 |  | 5 | 2.22 | 0.70 | 5.23 |  | 16 | **2.04** | **1.16** | **3.31** |
| Rheumatoid arthritis | 99 | **2.03** | **1.65** | **2.47** |  | 366 | **1.63** | **1.46** | **1.80** |  | 186 | **1.36** | **1.17** | **1.57** |  | 106 | **1.38** | **1.13** | **1.67** |  | 757 | **1.55** | **1.44** | **1.67** |
| Sarcoidosis | 2 | 0.50 | 0.05 | 1.82 |  | 33 | **1.50** | **1.03** | **2.11** |  | 17 | 0.94 | 0.55 | 1.51 |  | 22 | 1.10 | 0.69 | 1.66 |  | 74 | 1.15 | 0.91 | 1.45 |
| Sjögren´s syndrome | 1 | 2.13 | 0.00 | 12.20 |  | 2 | 0.84 | 0.08 | 3.10 |  | 1 | 0.52 | 0.00 | 2.99 |  | 2 | 1.83 | 0.17 | 6.75 |  | 6 | 1.03 | 0.37 | 2.25 |
| Systemic lupus erythematosus | 5 | 2.07 | 0.65 | 4.86 |  | 18 | **1.68** | **1.00** | **2.67** |  | 18 | **2.56** | **1.52** | **4.06** |  | 3 | 0.67 | 0.13 | 1.98 |  | 44 | **1.79** | **1.30** | **2.40** |
| Systemic sclerosis | 1 | 0.72 | 0.00 | 4.15 |  | 10 | 2.30 | 1.10 | 4.25 |  | 3 | 1.17 | 0.22 | 3.46 |  | 2 | 1.02 | 0.10 | 3.75 |  | 16 | 1.56 | 0.89 | 2.54 |
| Ulcerative colitis | 38 | **2.08** | **1.47** | **2.86** |  | 118 | 1.15 | 0.96 | 1.38 |  | 100 | 1.15 | 0.93 | 1.39 |  | 89 | 1.09 | 0.87 | 1.34 |  | 345 | **1.19** | **1.07** | **1.32** |
| Wegener´s granulomatosis | 3 | 0.83 | 0.16 | 2.47 |  | 6 | 0.46 | 0.16 | 1.00 |  | 14 | 1.69 | 0.92 | 2.84 |  | 8 | 1.84 | 0.79 | 3.65 |  | 31 | 1.05 | 0.72 | 1.50 |
| All | 340 | **1.79** | **1.61** | **1.99** |  | 1354 | **1.45** | **1.37** | **1.53** |  | 878 | **1.35** | **1.26** | **1.44** |  | 644 | **1.32** | **1.22** | **1.42** |  | 3216 | **1.42** | **1.37** | **1.47** |
| O = observed number of cases; SIR = standardized incidence ratio; CI = confidence interval. | | | | | | | | | |  |  |  |  |  |  |  |  |  |  |  |  |  |  |  |
| Bold type: 95% CI does not include 1.00.  Adjusted for age, period, socioeconomic status, region of residence, hospitalization of chronic lower respiratory diseases, obesity, alcoholism, hypertension, diabetes, atrial fibrillation, heart failure, renal disease, sepsis, and coronary heart disease. | | | | | | | | | | | | | | | | | | | | | | | | |

| **Supplement Table 8. SIR for subsequent ischemic stroke of female patients with IMD** | | | | | | | | | | | | | | | | | | | | | | | | |
| --- | --- | --- | --- | --- | --- | --- | --- | --- | --- | --- | --- | --- | --- | --- | --- | --- | --- | --- | --- | --- | --- | --- | --- | --- |
|  | Follow-up interval (years) | | | | | | | | | | | | | | | | | | |  |  |  |  |  |
|  | <1 | | | |  | 1-5 | | | |  | 5-10 | | | |  | >=10 | | | |  | All | | | |
| Immune-mediated diseases | O | SIR | 95% CI | |  | O | SIR | 95% CI | |  | O | SIR | 95% CI | |  | O | SIR | 95% CI | |  | O | SIR | 95% CI | |
| Addison´s disease | 8 | **2.60** | **1.11** | **5.14** |  | 17 | 1.25 | 0.73 | 2.01 |  | 21 | **2.30** | **1.42** | **3.52** |  | 7 | 1.03 | 0.41 | 2.13 |  | 53 | **1.62** | **1.22** | **2.13** |
| Amyotrophic lateral sclerosis | 1 | 0.17 | 0.00 | 0.95 |  | 5 | 1.15 | 0.36 | 2.70 |  | 1 | 0.69 | 0.00 | 3.95 |  | 0 |  |  |  |  | 7 | 0.56 | 0.22 | 1.17 |
| Ankylosing spondylitis | 3 | 2.17 | 0.41 | 6.44 |  | 11 | 1.42 | 0.70 | 2.54 |  | 9 | 1.40 | 0.64 | 2.67 |  | 10 | 1.13 | 0.54 | 2.09 |  | 33 | 1.35 | 0.93 | 1.90 |
| Autoimmune hemolytic anemia | 3 | 1.86 | 0.35 | 5.52 |  | 7 | 1.04 | 0.41 | 2.15 |  | 8 | 2.05 | 0.87 | 4.05 |  | 1 | 0.48 | 0.00 | 2.77 |  | 19 | 1.32 | 0.80 | 2.07 |
| Behcet´s disease | 1 | 9.09 | 0.00 | 52.11 |  | 1 | 1.47 | 0.00 | 8.43 |  | 0 |  |  |  |  | 1 | 3.57 | 0.00 | 20.47 |  | 3 | 1.83 | 0.34 | 5.41 |
| Celiac disease | 6 | 2.59 | 0.93 | 5.67 |  | 15 | 1.05 | 0.59 | 1.74 |  | 9 | 0.77 | 0.35 | 1.47 |  | 16 | 1.37 | 0.78 | 2.23 |  | 46 | 1.15 | 0.84 | 1.54 |
| Chorea minor | 0 |  |  |  |  | 1 | 3.57 | 0.00 | 20.47 |  | 0 |  |  |  |  | 0 |  |  |  |  | 1 | 1.89 | 0.00 | 10.82 |
| Crohn´s disease | 23 | **2.01** | **1.27** | **3.01** |  | 91 | **1.53** | **1.23** | **1.88** |  | 57 | 1.19 | 0.90 | 1.55 |  | 51 | 1.18 | 0.88 | 1.56 |  | 222 | **1.37** | **1.20** | **1.56** |
| Diabetes mellitus type I | 0 |  |  |  |  | 0 |  |  |  |  | 2 | 3.13 | 0.29 | 11.49 |  | 7 | **3.68** | **1.46** | **7.63** |  | 9 | **2.74** | **1.24** | **5.23** |
| Discoid lupus erythematosus | 2 | 3.64 | 0.34 | 13.37 |  | 2 | 0.88 | 0.08 | 3.25 |  | 1 | 0.66 | 0.00 | 3.80 |  | 2 | 1.42 | 0.13 | 5.22 |  | 7 | 1.22 | 0.48 | 2.53 |
| Grave´s disease | 88 | **2.35** | **1.89** | **2.90** |  | 313 | **1.35** | **1.21** | **1.51** |  | 292 | **1.40** | **1.25** | **1.57** |  | 227 | **1.26** | **1.10** | **1.43** |  | 920 | **1.40** | **1.31** | **1.49** |
| Hashimoto´s thyroiditis | 59 | **2.99** | **2.27** | **3.85** |  | 166 | **1.72** | **1.47** | **2.00** |  | 98 | **1.46** | **1.18** | **1.78** |  | 68 | **1.29** | **1.00** | **1.63** |  | 391 | **1.65** | **1.49** | **1.83** |
| Immune thrombocytopenic purpura | 9 | **2.59** | **1.17** | **4.93** |  | 23 | 1.35 | 0.86 | 2.03 |  | 10 | 0.81 | 0.39 | 1.50 |  | 8 | 0.97 | 0.42 | 1.93 |  | 50 | 1.22 | 0.90 | 1.61 |
| Localized scleroderma | 2 | 1.52 | 0.14 | 5.57 |  | 11 | 1.21 | 0.60 | 2.17 |  | 17 | **1.80** | **1.04** | **2.88** |  | 9 | 0.92 | 0.42 | 1.76 |  | 39 | 1.31 | 0.93 | 1.80 |
| Lupoid hepatitis | 2 | 3.77 | 0.36 | 13.88 |  | 1 | 0.69 | 0.00 | 3.98 |  | 0 |  |  |  |  | 0 |  |  |  |  | 3 | 1.24 | 0.23 | 3.67 |
| Multiple sclerosis | 25 | **3.46** | **2.24** | **5.12** |  | 43 | 1.16 | 0.84 | 1.56 |  | 34 | 1.15 | 0.79 | 1.60 |  | 21 | 0.88 | 0.55 | 1.35 |  | 123 | **1.26** | **1.04** | **1.50** |
| Myasthenia gravis | 2 | 0.73 | 0.07 | 2.69 |  | 20 | **1.63** | **1.00** | **2.53** |  | 10 | 1.21 | 0.58 | 2.24 |  | 7 | 1.20 | 0.48 | 2.48 |  | 39 | 1.34 | 0.95 | 1.84 |
| Pernicious anemia | 15 | **1.78** | **1.00** | **2.95** |  | 70 | **1.43** | **1.11** | **1.81** |  | 50 | **1.35** | **1.00** | **1.77** |  | 35 | 1.35 | 0.94 | 1.87 |  | 170 | **1.41** | **1.21** | **1.64** |
| Polyarteritis nodosa | 3 | 1.25 | 0.24 | 3.70 |  | 10 | 1.24 | 0.59 | 2.29 |  | 6 | 1.16 | 0.42 | 2.54 |  | 3 | 0.71 | 0.13 | 2.10 |  | 22 | 1.11 | 0.69 | 1.68 |
| Polymyalgia rheumatica | 120 | **1.94** | **1.61** | **2.32** |  | 515 | **1.51** | **1.38** | **1.64** |  | 346 | **1.45** | **1.31** | **1.62** |  | 228 | **1.52** | **1.33** | **1.73** |  | 1209 | **1.53** | **1.44** | **1.62** |
| Polymyositis/dermatomyositis | 9 | **4.97** | **2.25** | **9.48** |  | 9 | 1.35 | 0.61 | 2.58 |  | 4 | 1.27 | 0.33 | 3.27 |  | 3 | 1.78 | 0.33 | 5.25 |  | 25 | **1.88** | **1.21** | **2.77** |
| Primary biliary cirrhosis | 3 | 1.49 | 0.28 | 4.40 |  | 9 | 1.44 | 0.65 | 2.75 |  | 3 | 0.82 | 0.15 | 2.43 |  | 1 | 2.63 | 0.00 | 15.08 |  | 16 | 1.30 | 0.74 | 2.12 |
| Psoriasis | 21 | **1.82** | **1.12** | **2.79** |  | 117 | **1.72** | **1.42** | **2.06** |  | 84 | **1.48** | **1.18** | **1.83** |  | 74 | **1.39** | **1.09** | **1.75** |  | 296 | **1.56** | **1.39** | **1.75** |
| Reiter´s disease | 0 |  |  |  |  | 2 | 3.33 | 0.31 | 12.26 |  | 1 | 1.61 | 0.00 | 9.25 |  | 0 |  |  |  |  | 3 | 1.79 | 0.34 | 5.29 |
| Rheumatic fever | 3 | 4.35 | 0.82 | 12.87 |  | 7 | 2.09 | 0.83 | 4.33 |  | 8 | **3.51** | **1.50** | **6.95** |  | 2 | 1.23 | 0.12 | 4.54 |  | 20 | **2.52** | **1.54** | **3.90** |
| Rheumatoid arthritis | 246 | **2.10** | **1.84** | **2.37** |  | 900 | **1.67** | **1.56** | **1.78** |  | 477 | **1.49** | **1.36** | **1.63** |  | 220 | **1.27** | **1.11** | **1.45** |  | 1843 | **1.60** | **1.53** | **1.68** |
| Sarcoidosis | 7 | 1.34 | 0.53 | 2.77 |  | 37 | 1.38 | 0.97 | 1.90 |  | 34 | 1.24 | 0.86 | 1.73 |  | 34 | 1.06 | 0.74 | 1.49 |  | 112 | **1.22** | **1.01** | **1.47** |
| Sjögren´s syndrome | 9 | **2.63** | **1.19** | **5.02** |  | 26 | 1.45 | 0.95 | 2.13 |  | 14 | 1.02 | 0.56 | 1.72 |  | 13 | 1.20 | 0.63 | 2.05 |  | 62 | **1.35** | **1.04** | **1.73** |
| Systemic lupus erythematosus | 14 | **2.27** | **1.23** | **3.81** |  | 70 | **2.58** | **2.01** | **3.26** |  | 36 | **1.71** | **1.20** | **2.37** |  | 27 | 1.39 | 0.92 | 2.03 |  | 147 | **1.99** | **1.68** | **2.34** |
| Systemic sclerosis | 10 | **2.26** | **1.08** | **4.18** |  | 18 | 0.97 | 0.57 | 1.54 |  | 8 | 1.20 | 0.51 | 2.38 |  | 0 |  |  |  |  | 36 | 1.10 | 0.77 | 1.52 |
| Ulcerative colitis | 33 | **2.23** | **1.53** | **3.13** |  | 113 | **1.43** | **1.18** | **1.72** |  | 62 | 1.00 | 0.77 | 1.28 |  | 57 | 1.01 | 0.76 | 1.30 |  | 265 | **1.25** | **1.10** | **1.41** |
| Wegener´s granulomatosis | 8 | **2.64** | **1.13** | **5.23** |  | 6 | 0.49 | 0.18 | 1.07 |  | 12 | 1.39 | 0.72 | 2.44 |  | 4 | 1.45 | 0.38 | 3.75 |  | 30 | 1.13 | 0.76 | 1.61 |
| All | 735 | **2.15** | **1.99** | **2.31** |  | 2636 | **1.53** | **1.48** | **1.59** |  | 1714 | **1.40** | **1.33** | **1.47** |  | 1136 | **1.27** | **1.20** | **1.35** |  | 6221 | **1.49** | **1.45** | **1.53** |
| O = observed number of cases; SIR = standardized incidence ratio; CI = confidence interval. | | | | | | | | | |  |  |  |  |  |  |  |  |  |  |  |  |  |  |  |
| Bold type: 95% CI does not include 1.00.  Adjusted for age, period, socioeconomic status, region of residence, hospitalization of chronic lower respiratory diseases, obesity, alcoholism, hypertension, diabetes, atrial fibrillation, heart failure, renal disease, sepsis, and coronary heart disease. | | | | | | | | | | | | | | | | | | | | | | | | |

| **Supplement Table 9. SIR for subsequent ischemic stroke of patients with IMD** | | | | | | | | | | | | | | | | | | | | | | | | |
| --- | --- | --- | --- | --- | --- | --- | --- | --- | --- | --- | --- | --- | --- | --- | --- | --- | --- | --- | --- | --- | --- | --- | --- | --- |
|  | Follow-up interval (years) | | | | | | | | | | | | | | | | | | |  |  |  |  |  |
|  | <1 | | | |  | 1-5 | | | |  | 5-10 | | | |  | >=10 | | | |  | All | | | |
| Immune-mediated diseases | O | SIR | 95% CI | |  | O | SIR | 95% CI | |  | O | SIR | 95% CI | |  | O | SIR | 95% CI | |  | O | SIR | 95% CI | |
| Addison´s disease | 14 | **2.71** | **1.48** | **4.56** |  | 28 | 1.17 | 0.78 | 1.69 |  | 30 | **1.90** | **1.28** | **2.72** |  | 11 | 0.97 | 0.48 | 1.74 |  | 83 | **1.48** | **1.18** | **1.83** |
| Amyotrophic lateral sclerosis | 7 | 0.53 | 0.21 | 1.10 |  | 16 | 1.52 | 0.87 | 2.47 |  | 7 | 1.77 | 0.70 | 3.67 |  | 2 | 1.15 | 0.11 | 4.23 |  | 32 | 1.09 | 0.74 | 1.54 |
| Ankylosing spondylitis | 8 | 1.62 | 0.69 | 3.21 |  | 44 | **1.55** | **1.13** | **2.08** |  | 24 | 0.98 | 0.63 | 1.46 |  | 35 | 1.08 | 0.75 | 1.50 |  | 111 | **1.23** | **1.01** | **1.48** |
| Autoimmune hemolytic anemia | 4 | 1.45 | 0.38 | 3.75 |  | 12 | 1.00 | 0.51 | 1.75 |  | 19 | **2.51** | **1.51** | **3.93** |  | 5 | 1.23 | 0.39 | 2.90 |  | 40 | **1.51** | **1.08** | **2.06** |
| Behcet´s disease | 1 | 4.00 | 0.00 | 22.93 |  | 1 | 0.65 | 0.00 | 3.70 |  | 0 |  |  |  |  | 1 | 1.43 | 0.00 | 8.19 |  | 3 | 0.78 | 0.15 | 2.29 |
| Celiac disease | 9 | 2.17 | 0.99 | 4.14 |  | 29 | 1.28 | 0.86 | 1.84 |  | 21 | 1.17 | 0.72 | 1.78 |  | 26 | 1.47 | 0.96 | 2.15 |  | 85 | **1.36** | **1.09** | **1.68** |
| Chorea minor | 0 |  |  |  |  | 1 | 2.27 | 0.00 | 13.03 |  | 0 |  |  |  |  | 0 |  |  |  |  | 1 | 1.11 | 0.00 | 6.37 |
| Crohn disease | 49 | **2.15** | **1.59** | **2.84** |  | 160 | **1.33** | **1.13** | **1.55** |  | 103 | 1.11 | 0.91 | 1.35 |  | 97 | 1.15 | 0.93 | 1.40 |  | 409 | **1.28** | **1.16** | **1.41** |
| Diabetes mellitus type I | 1 | 6.25 | 0.00 | 35.83 |  | 2 | 0.45 | 0.04 | 1.65 |  | 5 | 2.75 | 0.87 | 6.46 |  | 17 | **5.00** | **2.91** | **8.02** |  | 25 | **2.54** | **1.64** | **3.76** |
| Discoid lupus erythematosus | 3 | 4.23 | 0.80 | 12.51 |  | 3 | 0.99 | 0.19 | 2.92 |  | 1 | 0.47 | 0.00 | 2.70 |  | 3 | 1.55 | 0.29 | 4.60 |  | 10 | 1.28 | 0.61 | 2.37 |
| Grave´s disease | 101 | **2.15** | **1.76** | **2.62** |  | 402 | **1.39** | **1.26** | **1.53** |  | 348 | **1.36** | **1.22** | **1.51** |  | 276 | **1.27** | **1.12** | **1.43** |  | 1127 | **1.39** | **1.31** | **1.48** |
| Hashimoto´s thyroiditis | 77 | **2.99** | **2.36** | **3.74** |  | 211 | **1.73** | **1.50** | **1.98** |  | 115 | **1.39** | **1.14** | **1.67** |  | 82 | **1.28** | **1.02** | **1.59** |  | 485 | **1.64** | **1.50** | **1.80** |
| Immune thrombocytopenic purpura | 16 | **2.35** | **1.34** | **3.83** |  | 55 | **1.77** | **1.33** | **2.30** |  | 19 | 0.94 | 0.57 | 1.48 |  | 14 | 1.20 | 0.65 | 2.01 |  | 104 | **1.49** | **1.22** | **1.81** |
| Localized scleroderma | 2 | 1.28 | 0.12 | 4.71 |  | 13 | 1.25 | 0.66 | 2.14 |  | 18 | **1.72** | **1.01** | **2.72** |  | 11 | 1.04 | 0.51 | 1.86 |  | 44 | 1.33 | 0.97 | 1.79 |
| Lupoid hepatitis | 3 | 4.48 | 0.84 | 13.25 |  | 4 | 1.98 | 0.52 | 5.12 |  | 0 |  |  |  |  | 0 |  |  |  |  | 7 | 2.10 | 0.83 | 4.36 |
| Multiple sclerosis | 40 | **3.05** | **2.18** | **4.15** |  | 73 | 1.09 | 0.85 | 1.37 |  | 55 | 1.11 | 0.83 | 1.44 |  | 35 | 0.95 | 0.66 | 1.32 |  | 203 | **1.22** | **1.06** | **1.40** |
| Myasthenia gravis | 6 | 1.01 | 0.36 | 2.21 |  | 38 | 1.36 | 0.96 | 1.87 |  | 23 | 1.20 | 0.76 | 1.80 |  | 13 | 1.08 | 0.57 | 1.85 |  | 80 | 1.23 | 0.97 | 1.53 |
| Pernicious anemia | 25 | **1.56** | **1.01** | **2.31** |  | 138 | **1.49** | **1.25** | **1.76** |  | 89 | 1.23 | 0.99 | 1.52 |  | 74 | **1.44** | **1.13** | **1.80** |  | 326 | **1.40** | **1.25** | **1.56** |
| Polyarteritis nodosa | 5 | 1.23 | 0.39 | 2.89 |  | 20 | 1.30 | 0.79 | 2.02 |  | 11 | 1.05 | 0.52 | 1.88 |  | 10 | 1.13 | 0.54 | 2.08 |  | 46 | 1.19 | 0.87 | 1.58 |
| Polymyalgia rheumatica | 165 | **1.76** | **1.50** | **2.05** |  | 761 | **1.50** | **1.39** | **1.61** |  | 529 | **1.54** | **1.41** | **1.68** |  | 322 | **1.53** | **1.37** | **1.71** |  | 1777 | **1.54** | **1.47** | **1.61** |
| Polymyositis/dermatomyositis | 10 | **3.46** | **1.65** | **6.39** |  | 13 | 1.19 | 0.63 | 2.03 |  | 6 | 1.07 | 0.38 | 2.34 |  | 5 | 1.73 | 0.55 | 4.07 |  | 34 | **1.52** | **1.05** | **2.13** |
| Primary biliary cirrhosis | 4 | 1.54 | 0.40 | 3.98 |  | 11 | 1.45 | 0.72 | 2.60 |  | 4 | 0.91 | 0.24 | 2.35 |  | 1 | 2.17 | 0.00 | 12.46 |  | 20 | 1.33 | 0.81 | 2.05 |
| Psoriasis | 44 | **1.92** | **1.39** | **2.58** |  | 217 | **1.65** | **1.44** | **1.89** |  | 163 | **1.53** | **1.30** | **1.78** |  | 144 | **1.41** | **1.19** | **1.66** |  | 568 | **1.56** | **1.44** | **1.70** |
| Reiter´s disease | 0 |  |  |  |  | 5 | 2.02 | 0.64 | 4.76 |  | 6 | 2.47 | 0.89 | 5.41 |  | 2 | 0.56 | 0.05 | 2.07 |  | 13 | 1.47 | 0.78 | 2.52 |
| Rheumatic fever | 5 | **3.91** | **1.23** | **9.19** |  | 10 | 1.66 | 0.79 | 3.06 |  | 14 | **3.04** | **1.65** | **5.11** |  | 7 | 1.81 | 0.72 | 3.75 |  | 36 | **2.28** | **1.59** | **3.16** |
| Rheumatoid arthritis | 345 | **2.08** | **1.86** | **2.31** |  | 1266 | **1.66** | **1.57** | **1.75** |  | 663 | **1.45** | **1.34** | **1.56** |  | 326 | **1.30** | **1.16** | **1.45** |  | 2600 | **1.59** | **1.53** | **1.65** |
| Sarcoidosis | 9 | 0.97 | 0.44 | 1.85 |  | 70 | **1.43** | **1.12** | **1.81** |  | 51 | 1.12 | 0.83 | 1.47 |  | 56 | 1.08 | 0.81 | 1.40 |  | 186 | **1.19** | **1.03** | **1.38** |
| Sjögren´s syndrome | 10 | **2.57** | **1.22** | **4.75** |  | 28 | 1.38 | 0.92 | 1.99 |  | 15 | 0.96 | 0.54 | 1.59 |  | 15 | 1.26 | 0.70 | 2.08 |  | 68 | **1.31** | **1.02** | **1.67** |
| Systemic lupus erythematosus | 19 | **2.21** | **1.33** | **3.46** |  | 88 | **2.33** | **1.87** | **2.87** |  | 54 | **1.92** | **1.44** | **2.51** |  | 30 | 1.26 | 0.85 | 1.80 |  | 191 | **1.94** | **1.68** | **2.24** |
| Systemic sclerosis | 11 | 1.90 | 0.94 | 3.41 |  | 28 | 1.22 | 0.81 | 1.77 |  | 11 | 1.19 | 0.59 | 2.14 |  | 2 | 0.39 | 0.04 | 1.43 |  | 52 | 1.21 | 0.90 | 1.58 |
| Ulcerative colitis | 71 | **2.15** | **1.68** | **2.71** |  | 231 | **1.27** | **1.11** | **1.45** |  | 162 | 1.09 | 0.92 | 1.27 |  | 146 | 1.05 | 0.89 | 1.24 |  | 610 | **1.21** | **1.12** | **1.31** |
| Wegener´s granulomatosis | 11 | 1.66 | 0.82 | 2.98 |  | 12 | 0.47 | 0.24 | 0.83 |  | 26 | **1.54** | **1.00** | **2.25** |  | 12 | 1.69 | 0.87 | 2.96 |  | 61 | 1.09 | 0.83 | 1.40 |
| All | 1075 | **2.02** | **1.90** | **2.14** |  | 3990 | **1.50** | **1.46** | **1.55** |  | 2592 | **1.38** | **1.33** | **1.43** |  | 1780 | **1.29** | **1.23** | **1.35** |  | 9437 | **1.46** | **1.43** | **1.49** |
| O = observed number of cases; SIR = standardized incidence ratio; CI = confidence interval. | | | | | | | | | |  |  |  |  |  |  |  |  |  |  |  |  |  |  |  |
| Bold type: 95% CI does not include 1.00.  Adjusted for age, period, socioeconomic status, region of residence, hospitalization of chronic lower respiratory diseases, obesity, alcoholism, hypertension, diabetes, atrial fibrillation, heart failure, renal disease, sepsis, and coronary heart disease. | | | | | | | | | | | | | | | | | | | | | | | | |

| **Supplement Table 10. SIR for subsequent ischemic stroke of male patients with IMD after one year of follow-up** | | | | | | | | | | | | | | | | |  |  |  |
| --- | --- | --- | --- | --- | --- | --- | --- | --- | --- | --- | --- | --- | --- | --- | --- | --- | --- | --- | --- |
|  | Age at diagnosis of ischemic stroke (years) | | | | | | | | | | | | | | | | | | |
|  | <50 | | | |  | 50-59 | | | |  | 60-69 | | | |  | >=70 | | | |
| Immune-mediated diseases | O | SIR | 95% CI | |  | O | SIR | 95% CI | |  | O | SIR | 95% CI | |  | O | SIR | 95% CI | |
| Addison´s disease | 0 |  |  |  |  | 1 | 0.66 | 0.00 | 3.77 |  | 8 | **2.52** | **1.08** | **5.00** |  | 15 | 0.95 | 0.53 | 1.57 |
| Amyotrophic lateral sclerosis | 1 | 25.00 | 0.01 | 143.31 |  | 1 | 2.86 | 0.00 | 16.38 |  | 5 | 2.69 | 0.85 | 6.32 |  | 12 | 1.59 | 0.82 | 2.78 |
| Ankylosing spondylitis | 0 | 0.00 | 0.32 | 1.29 |  | 16 | 1.40 | 0.80 | 2.27 |  | 28 | 1.34 | 0.89 | 1.95 |  | 29 | 1.07 | 0.72 | 1.54 |
| Autoimmune hemolytic anemia | 2 | **14.29** | **1.35** | **52.54** |  | 0 |  |  |  |  | 4 | 2.76 | 0.72 | 7.13 |  | 14 | 1.55 | 0.84 | 2.61 |
| Behcet´s disease | 0 |  |  |  |  | 0 |  |  |  |  | 0 |  |  |  |  | 0 |  |  |  |
| Celiac disease | 5 | **3.79** | **1.20** | **8.91** |  | 5 | 2.65 | 0.83 | 6.22 |  | 8 | 1.72 | 0.74 | 3.41 |  | 18 | 1.39 | 0.82 | 2.21 |
| Chorea minor | 0 |  |  |  |  | 0 |  |  |  |  | 0 |  |  |  |  | 0 |  |  |  |
| Crohn´s disease | 7 | 0.93 | 0.37 | 1.94 |  | 22 | 1.08 | 0.67 | 1.63 |  | 45 | 1.10 | 0.81 | 1.48 |  | 87 | 1.11 | 0.89 | 1.38 |
| Diabetes mellitus type I | 15 | **2.32** | **1.29** | **3.83** |  | 0 |  |  |  |  | 0 |  |  |  |  | 0 |  |  |  |
| Discoid lupus erythematosus | 0 |  |  |  |  | 0 |  |  |  |  | 1 | 1.96 | 0.00 | 11.24 |  | 1 | 0.82 | 0.00 | 4.70 |
| Grave´s disease | 1 | 0.35 | 0.00 | 1.98 |  | 17 | **2.01** | **1.17** | **3.23** |  | 41 | **1.43** | **1.03** | **1.94** |  | 135 | **1.32** | **1.10** | **1.56** |
| Hashimoto´s thyroiditis | 0 |  |  |  |  | 6 | 2.32 | 0.83 | 5.08 |  | 7 | 0.89 | 0.35 | 1.84 |  | 63 | **1.51** | **1.16** | **1.94** |
| Immune thrombocytopenic purpura | 5 | **6.67** | **2.10** | **15.68** |  | 4 | 2.86 | 0.74 | 7.39 |  | 7 | 1.39 | 0.55 | 2.89 |  | 31 | **1.70** | **1.15** | **2.42** |
| Localized scleroderma | 0 |  |  |  |  | 0 |  |  |  |  | 0 |  |  |  |  | 5 | 2.07 | 0.65 | 4.86 |
| Lupoid hepatitis | 0 |  |  |  |  | 0 |  |  |  |  | 1 | 5.26 | 0.00 | 30.17 |  | 2 | 5.56 | 0.52 | 20.43 |
| Multiple sclerosis | 2 | 0.69 | 0.07 | 2.55 |  | 18 | **2.41** | **1.43** | **3.82** |  | 18 | 0.97 | 0.57 | 1.53 |  | 27 | 0.80 | 0.52 | 1.16 |
| Myasthenia gravis | 0 |  |  |  |  | 1 | 1.11 | 0.00 | 6.37 |  | 7 | 1.46 | 0.58 | 3.02 |  | 29 | 1.08 | 0.72 | 1.55 |
| Pernicious anemia | 0 |  |  |  |  | 1 | 0.72 | 0.00 | 4.15 |  | 6 | 0.72 | 0.26 | 1.58 |  | 139 | **1.47** | **1.24** | **1.74** |
| Polyarteritis nodosa | 0 |  |  |  |  | 1 | 0.93 | 0.00 | 5.36 |  | 8 | 2.05 | 0.87 | 4.05 |  | 13 | 1.08 | 0.57 | 1.85 |
| Polymyalgia rheumatica | 0 |  |  |  |  | 3 | 1.33 | 0.25 | 3.95 |  | 43 | **1.70** | **1.23** | **2.30** |  | 477 | **1.57** | **1.43** | **1.71** |
| Polymyositis/dermatomyositis | 1 | 10.00 | 0.00 | 57.32 |  | 2 | 3.45 | 0.33 | 12.68 |  | 1 | 0.57 | 0.00 | 3.26 |  | 4 | 0.72 | 0.19 | 1.87 |
| Primary biliary cirrhosis | 0 |  |  |  |  | 0 |  |  |  |  | 1 | 1.08 | 0.00 | 6.16 |  | 2 | 1.79 | 0.17 | 6.57 |
| Psoriasis | 8 | 1.85 | 0.79 | 3.66 |  | 27 | **1.52** | **1.00** | **2.21** |  | 58 | 1.31 | 0.99 | 1.69 |  | 156 | **1.63** | **1.39** | **1.91** |
| Reiter´s disease | 1 | 1.82 | 0.00 | 10.42 |  | 1 | 0.68 | 0.00 | 3.93 |  | 1 | 0.46 | 0.00 | 2.62 |  | 7 | **2.60** | **1.03** | **5.39** |
| Rheumatic fever | 0 |  |  |  |  | 2 | 3.17 | 0.30 | 11.67 |  | 1 | 0.68 | 0.00 | 3.90 |  | 11 | **2.23** | **1.11** | **4.01** |
| Rheumatoid arthritis | 7 | **3.02** | **1.20** | **6.25** |  | 23 | 1.48 | 0.94 | 2.23 |  | 112 | **1.38** | **1.14** | **1.66** |  | 516 | **1.52** | **1.39** | **1.65** |
| Sarcoidosis | 3 | 0.72 | 0.14 | 2.13 |  | 13 | 1.38 | 0.73 | 2.36 |  | 23 | 1.38 | 0.88 | 2.08 |  | 33 | 1.10 | 0.76 | 1.55 |
| Sjögren´s syndrome | 0 |  |  |  |  | 0 |  |  |  |  | 2 | 1.61 | 0.15 | 5.93 |  | 3 | 0.77 | 0.15 | 2.29 |
| Systemic lupus erythematosus | 2 | 1.31 | 0.12 | 4.81 |  | 4 | 2.65 | 0.69 | 6.85 |  | 12 | **2.23** | **1.15** | **3.92** |  | 21 | 1.52 | 0.94 | 2.33 |
| Systemic sclerosis | 2 | **13.33** | **1.26** | **49.03** |  | 2 | 2.35 | 0.22 | 8.65 |  | 6 | **3.61** | **1.30** | **7.92** |  | 5 | 0.80 | 0.25 | 1.89 |
| Ulcerative colitis | 10 | 0.80 | 0.38 | 1.47 |  | 33 | 0.98 | 0.67 | 1.37 |  | 70 | 1.05 | 0.82 | 1.33 |  | 194 | **1.22** | **1.06** | **1.41** |
| Wegener´s granulomatosis | 1 | 2.94 | 0.00 | 16.86 |  | 2 | 1.11 | 0.10 | 4.09 |  | 2 | 0.37 | 0.03 | 1.34 |  | 23 | 1.26 | 0.80 | 1.90 |
| All | 73 | **1.35** | **1.06** | **1.70** |  | 205 | **1.41** | **1.22** | **1.61** |  | 526 | **1.30** | **1.19** | **1.41** |  | 2072 | **1.41** | **1.35** | **1.47** |
| O = observed number of cases; SIR = standardized incidence ratio; CI = confidence interval. | | | | | | | | | |  |  |  |  |  |  |  |  |  |  |
| Bold type: 95% CI does not include 1.00.  Adjusted for age, period, socioeconomic status, region of residence, hospitalization of chronic lower respiratory diseases, obesity, alcoholism, hypertension, diabetes, atrial fibrillation, heart failure, renal disease, sepsis, and coronary heart disease | | | | | | | | | | | | | | | | | | | |

| **Supplement Table 11. SIR for subsequent ischemic stroke of female patients with IMD after one year of follow-up** | | | | | | | | | | | | | | | | |  |  |  |  |
| --- | --- | --- | --- | --- | --- | --- | --- | --- | --- | --- | --- | --- | --- | --- | --- | --- | --- | --- | --- | --- |
|  | Age at diagnosis of ischemic stroke (years) | | | | | | | | | | | | | | | | | | |  |
|  | <50 | | | |  | 50-59 | | | |  | 60-69 | | | |  | >=70 | | | |  |
| Immune-mediated diseases | O | SIR | 95% CI | |  | O | SIR | 95% CI | |  | O | SIR | 95% CI | |  | O | SIR | 95% CI | |  |
| Addison´s disease | 0 |  |  |  |  | 1 | 0.55 | 0.00 | 3.15 |  | 6 | 1.53 | 0.55 | 3.35 |  | 38 | **1.65** | **1.17** | **2.27** |  |
| Amyotrophic lateral sclerosis | 0 |  |  |  |  | 0 |  |  |  |  | 2 | 3.57 | 0.34 | 13.13 |  | 4 | 0.70 | 0.18 | 1.82 |  |
| Ankylosing spondylitis | 3 | 4.05 | 0.76 | 12.00 |  | 7 | 2.46 | 0.97 | 5.09 |  | 6 | 1.09 | 0.39 | 2.39 |  | 14 | 1.01 | 0.55 | 1.69 |  |
| Autoimmune hemolytic anemia | 0 |  |  |  |  |  |  |  |  |  | 1 | 1.35 | 0.00 | 7.75 |  | 15 | 1.28 | 0.71 | 2.12 |  |
| Behcet´s disease | 0 |  |  |  |  | 1 | 4.55 | 0.00 | 26.06 |  | 0 |  |  |  |  | 1 | 0.99 | 0.00 | 5.68 |  |
| Celiac disease | 0 |  |  |  |  | 5 | 2.54 | 0.80 | 5.97 |  | 7 | 1.52 | 0.60 | 3.15 |  | 28 | 0.96 | 0.64 | 1.39 |  |
| Chorea minor | 0 |  |  |  |  | 0 |  |  |  |  | 0 |  |  |  |  | 1 | 2.70 | 0.00 | 15.49 |  |
| Crohn´s disease | 13 | **2.13** | **1.13** | **3.65** |  | 22 | **1.75** | **1.10** | **2.66** |  | 34 | 1.25 | 0.86 | 1.74 |  | 130 | **1.25** | **1.04** | **1.48** |  |
| Diabetes mellitus type I | 9 | **2.80** | **1.27** | **5.35** |  | 0 |  |  |  |  | 0 |  |  |  |  | 0 |  |  |  |  |
| Discoid lupus erythematosus | 0 |  |  |  |  | 1 | 2.86 | 0.00 | 16.38 |  | 0 |  |  |  |  | 4 | 1.03 | 0.27 | 2.67 |  |
| Grave´s disease | 18 | 1.45 | 0.86 | 2.30 |  | 26 | 1.00 | 0.65 | 1.47 |  | 96 | 1.20 | 0.97 | 1.46 |  | 692 | **1.38** | **1.28** | **1.49** |  |
| Hashimoto´s thyroiditis | 6 | **3.95** | **1.42** | **8.65** |  | 5 | 0.82 | 0.26 | 1.93 |  | 34 | **1.63** | **1.13** | **2.28** |  | 287 | **1.53** | **1.35** | **1.71** |  |
| Immune thrombocytopenic purpura | 3 | 4.41 | 0.83 | 13.06 |  | 5 | **4.90** | **1.55** | **11.53** |  | 4 | 1.27 | 0.33 | 3.29 |  | 29 | 0.89 | 0.59 | 1.27 |  |
| Localized scleroderma | 0 |  |  |  |  | 2 | 6.25 | 0.59 | 22.99 |  | 4 | 2.09 | 0.54 | 5.42 |  | 31 | 1.19 | 0.81 | 1.69 |  |
| Lupoid hepatitis | 0 |  |  |  |  | 0 |  |  |  |  | 1 | 2.38 | 0.00 | 13.65 |  | 0 |  |  |  |  |
| Multiple sclerosis | 8 | **2.57** | **1.10** | **5.09** |  | 11 | 1.12 | 0.56 | 2.01 |  | 20 | 1.01 | 0.62 | 1.57 |  | 59 | 1.02 | 0.77 | 1.31 |  |
| Myasthenia gravis | 0 |  |  |  |  | 2 | 1.96 | 0.18 | 7.21 |  | 4 | 1.48 | 0.39 | 3.83 |  | 31 | 1.40 | 0.95 | 1.99 |  |
| Pernicious anemia | 1 | 5.00 | 0.00 | 28.66 |  | 3 | 4.41 | 0.83 | 13.06 |  | 4 | 1.12 | 0.29 | 2.91 |  | 147 | **1.36** | **1.15** | **1.60** |  |
| Polyarteritis nodosa | 1 | 6.25 | 0.00 | 35.83 |  | 0 |  |  |  |  | 2 | 1.30 | 0.12 | 4.78 |  | 16 | 1.05 | 0.60 | 1.71 |  |
| Polymyalgia rheumatica | 0 |  |  |  |  | 6 | **2.86** | **1.03** | **6.26** |  | 34 | 1.35 | 0.93 | 1.89 |  | 1049 | **1.49** | **1.41** | **1.59** |  |
| Polymyositis/dermatomyositis | 1 | 8.33 | 0.00 | 47.77 |  | 1 | 1.56 | 0.00 | 8.96 |  | 3 | 2.22 | 0.42 | 6.58 |  | 11 | 1.17 | 0.58 | 2.10 |  |
| Primary biliary cirrhosis | 0 |  |  |  |  | 0 |  |  |  |  | 5 | 1.94 | 0.61 | 4.56 |  | 8 | 1.14 | 0.49 | 2.27 |  |
| Psoriasis | 8 | **3.31** | **1.41** | **6.55** |  | 10 | 1.03 | 0.49 | 1.90 |  | 45 | **1.85** | **1.35** | **2.48** |  | 212 | **1.50** | **1.30** | **1.71** |  |
| Reiter´s disease | 0 |  |  |  |  | 0 |  |  |  |  | 2 | 8.70 | 0.82 | 31.98 |  | 1 | 0.81 | 0.00 | 4.62 |  |
| Rheumatic fever | 0 |  |  |  |  | 1 | 6.25 | 0.00 | 35.83 |  | 0 |  |  |  |  | 16 | **2.46** | **1.40** | **4.01** |  |
| Rheumatoid arthritis | 8 | 1.72 | 0.73 | 3.41 |  | 29 | 1.40 | 0.93 | 2.01 |  | 172 | **1.72** | **1.47** | **2.00** |  | 1388 | **1.53** | **1.45** | **1.61** |  |
| Sarcoidosis | 4 | 2.80 | 0.73 | 7.23 |  | 11 | **2.55** | **1.27** | **4.58** |  | 16 | 1.06 | 0.60 | 1.72 |  | 74 | 1.13 | 0.89 | 1.42 |  |
| Sjögren´s syndrome | 0 |  |  |  |  | 4 | 2.25 | 0.58 | 5.81 |  | 6 | 1.00 | 0.36 | 2.20 |  | 43 | 1.25 | 0.90 | 1.68 |  |
| Systemic lupus erythematosus | 14 | **5.91** | **3.22** | **9.94** |  | 16 | **2.19** | **1.25** | **3.56** |  | 29 | **1.84** | **1.23** | **2.65** |  | 74 | **1.76** | **1.38** | **2.21** |  |
| Systemic sclerosis | 1 | 3.13 | 0.00 | 17.91 |  | 5 | **4.35** | **1.37** | **10.23** |  | 7 | 2.46 | 0.98 | 5.11 |  | 13 | 0.54 | 0.29 | 0.93 |  |
| Ulcerative colitis | 13 | 1.75 | 0.93 | 3.00 |  | 13 | 0.88 | 0.47 | 1.51 |  | 25 | 0.91 | 0.59 | 1.35 |  | 181 | **1.22** | **1.05** | **1.41** |  |
| Wegener´s granulomatosis | 0 |  |  |  |  | 1 | 1.56 | 0.00 | 8.96 |  | 2 | 0.78 | 0.07 | 2.87 |  | 19 | 0.94 | 0.56 | 1.47 |  |
| All | 111 | **2.17** | **1.78** | **2.61** |  | 188 | **1.45** | **1.25** | **1.67** |  | 571 | **1.42** | **1.31** | **1.54** |  | 4616 | **1.42** | **1.38** | **1.46** |  |
| O = observed number of cases; SIR = standardized incidence ratio; CI = confidence interval. | | | | | | | | | |  |  |  |  |  |  |  |  |  |  |  |
| Bold type: 95% CI does not include 1.00.  Adjusted for age, period, socioeconomic status, region of residence, hospitalization of chronic lower respiratory diseases, obesity, alcoholism, hypertension, diabetes, atrial fibrillation, heart failure, renal disease, sepsis, and coronary heart disease | | | | | | | | | | | | | | | | | | | |  |

| **Supplement Table 12. SIR for subsequent hemorrhagic stroke of patients with IMD after one year of follow-up** | | | | | | | | | |  |
| --- | --- | --- | --- | --- | --- | --- | --- | --- | --- | --- |
|  | Period of diagnosis (years) | | | | | | | | |  |
|  | 1987-1996 | | | |  | 1997-2008 | | | |  |
| Immune-mediated diseases | O | SIR | 95% CI | |  | O | SIR | 95% CI | |  |
| Addison´s disease | 0 |  |  |  |  | 4 | 0.68 | 0.18 | 1.75 |  |
| Amyotrophic lateral sclerosis | 0 |  |  |  |  | 3 | 1.81 | 0.34 | 5.35 |  |
| Ankylosing spondylitis | 12 | **4.84** | **2.49** | **8.48** |  | 24 | **1.96** | **1.25** | **2.92** |  |
| Autoimmune hemolytic anemia | 3 | 3.16 | 0.60 | 9.35 |  | 4 | 2.05 | 0.53 | 5.30 |  |
| Behcet´s disease | 0 |  |  |  |  | 0 |  |  |  |  |
| Celiac disease | 5 | 2.49 | 0.78 | 5.85 |  | 16 | **2.46** | **1.40** | **4.01** |  |
| Chorea minor | 0 |  |  |  |  | 0 |  |  |  |  |
| Crohn´s disease | 19 | **2.56** | **1.54** | **4.01** |  | 71 | **1.70** | **1.32** | **2.14** |  |
| Diabetes mellitus type I | 1 | 2.17 | 0.00 | 12.46 |  | 4 | 1.25 | 0.32 | 3.22 |  |
| Discoid lupus erythematosus | 0 |  |  |  |  | 1 | 1.41 | 0.00 | 8.07 |  |
| Grave´s disease | 33 | **1.60** | **1.10** | **2.25** |  | 114 | **1.64** | **1.35** | **1.97** |  |
| Hashimoto´s thyroiditis | 22 | **2.36** | **1.48** | **3.58** |  | 35 | **1.65** | **1.15** | **2.30** |  |
| Immune thrombocytopenic purpura | 3 | 2.56 | 0.48 | 7.59 |  | 17 | **2.28** | **1.32** | **3.66** |  |
| Localized scleroderma | 1 | 0.92 | 0.00 | 5.26 |  | 7 | **2.77** | **1.10** | **5.73** |  |
| Lupoid hepatitis | 0 |  |  |  |  | 0 |  |  |  |  |
| Multiple sclerosis | 6 | 1.16 | 0.42 | 2.55 |  | 24 | 1.17 | 0.75 | 1.75 |  |
| Myasthenia gravis | 7 | **4.79** | **1.90** | **9.93** |  | 7 | 1.00 | 0.40 | 2.07 |  |
| Pernicious anemia | 17 | 1.51 | 0.88 | 2.42 |  | 21 | 1.39 | 0.86 | 2.13 |  |
| Polyarteritis nodosa | 0 |  |  |  |  | 3 | 0.84 | 0.16 | 2.49 |  |
| Polymyalgia rheumatica | 53 | **1.66** | **1.24** | **2.17** |  | 130 | **1.46** | **1.22** | **1.74** |  |
| Polymyositis/dermatomyositis | 1 | 2.78 | 0.00 | 15.92 |  | 4 | 1.59 | 0.41 | 4.12 |  |
| Primary biliary cirrhosis | 0 |  |  |  |  | 5 | 1.83 | 0.58 | 4.31 |  |
| Psoriasis | 22 | **1.83** | **1.14** | **2.77** |  | 54 | **1.47** | **1.11** | **1.93** |  |
| Reiter´s disease | 1 | 3.33 | 0.00 | 19.11 |  | 1 | 0.93 | 0.00 | 5.36 |  |
| Rheumatic fever | 0 |  |  |  |  | 1 | 0.78 | 0.00 | 4.48 |  |
| Rheumatoid arthritis | 101 | **2.45** | **2.00** | **2.98** |  | 260 | **1.81** | **1.59** | **2.04** |  |
| Sarcoidosis | 11 | **2.75** | **1.36** | **4.94** |  | 21 | 1.30 | 0.80 | 1.99 |  |
| Sjögren´s syndrome | 1 | 1.14 | 0.00 | 6.51 |  | 4 | 0.83 | 0.21 | 2.14 |  |
| Systemic lupus erythematosus | 4 | 1.78 | 0.46 | 4.60 |  | 19 | **2.15** | **1.29** | **3.37** |  |
| Systemic sclerosis | 2 | 3.77 | 0.36 | 13.88 |  | 8 | **2.64** | **1.13** | **5.23** |  |
| Ulcerative colitis | 20 | 1.56 | 0.95 | 2.42 |  | 81 | **1.33** | **1.05** | **1.65** |  |
| Wegener´s granulomatosis | 1 | 1.16 | 0.00 | 6.67 |  | 4 | 0.71 | 0.19 | 1.85 |  |
| All | 346 | **1.98** | **1.78** | **2.20** |  | 947 | **1.58** | **1.48** | **1.69** |  |
| O = observed number of cases; SIR = standardized incidence ratio; CI = confidence interval. | | | | | | | | | |  |
| Bold type: 95% CI does not include 1.00. |  |  |  |  |  |  |  |  |  |  |
| Adjusted for age, period, socioeconomic status, region of residence, hospitalization of chronic lower respiratory diseases, obesity, alcoholism, hypertension, diabetes, atrial fibrillation, heart failure, renal disease, sepsis, and coronary heart disease. | | | | | | | | | | |

| **Supplement Table 13. SIR for subsequent ischemic stroke of patients with IMD after one year of follow-up** | | | | | | | | | | |
| --- | --- | --- | --- | --- | --- | --- | --- | --- | --- | --- |
|  | Period of diagnosis (years) | | | | | | | | |  |
|  | 1987-1996 | | | |  | 1997-2008 | | | |  |
| Immune-mediated diseases | O | SIR | 95% CI | |  | O | SIR | 95% CI | |  |
| Addison´s disease | 20 | 1.53 | 0.93 | 2.36 |  | 49 | 1.29 | 0.96 | 1.71 |  |
| Amyotrophic lateral sclerosis | 9 | 1.68 | 0.76 | 3.20 |  | 16 | 1.47 | 0.84 | 2.40 |  |
| Ankylosing spondylitis | 23 | 1.21 | 0.77 | 1.82 |  | 80 | 1.21 | 0.96 | 1.50 |  |
| Autoimmune hemolytic anemia | 15 | 1.58 | 0.88 | 2.62 |  | 21 | 1.48 | 0.91 | 2.27 |  |
| Behcet´s disease | 0 |  |  |  |  | 2 | 0.63 | 0.06 | 2.33 |  |
| Celiac disease | 18 | 1.14 | 0.68 | 1.81 |  | 58 | **1.36** | **1.03** | **1.76** |  |
| Chorea minor | 0 |  |  |  |  | 1 | 1.75 | 0.00 | 10.06 |  |
| Crohn´s disease | 79 | **1.41** | **1.12** | **1.76** |  | 281 | **1.17** | **1.03** | **1.31** |  |
| Diabetes mellitus type I | 3 | 3.57 | 0.67 | 10.57 |  | 21 | **2.38** | **1.47** | **3.64** |  |
| Discoid lupus erythematosus | 2 | 0.88 | 0.08 | 3.24 |  | 5 | 1.04 | 0.33 | 2.44 |  |
| Grave´s disease | 306 | **1.54** | **1.37** | **1.72** |  | 720 | **1.28** | **1.18** | **1.37** |  |
| Hashimoto´s thyroiditis | 177 | **1.82** | **1.56** | **2.11** |  | 231 | **1.34** | **1.18** | **1.53** |  |
| Immune thrombocytopenic purpura | 19 | **1.94** | **1.16** | **3.03** |  | 69 | **1.30** | **1.01** | **1.64** |  |
| Localized scleroderma | 14 | 1.25 | 0.68 | 2.10 |  | 28 | 1.38 | 0.91 | 1.99 |  |
| Lupoid hepatitis | 0 |  |  |  |  | 4 | 1.53 | 0.40 | 3.96 |  |
| Multiple sclerosis | 40 | 1.10 | 0.78 | 1.49 |  | 123 | 1.05 | 0.87 | 1.25 |  |
| Myasthenia gravis | 19 | 1.48 | 0.89 | 2.32 |  | 55 | 1.19 | 0.89 | 1.55 |  |
| Pernicious anemia | 148 | **1.44** | **1.22** | **1.69** |  | 153 | **1.35** | **1.14** | **1.58** |  |
| Polyarteritis nodosa | 8 | 0.85 | 0.36 | 1.68 |  | 33 | 1.31 | 0.90 | 1.84 |  |
| Polymyalgia rheumatica | 477 | **1.44** | **1.31** | **1.57** |  | 1135 | **1.56** | **1.47** | **1.65** |  |
| Polymyositis/dermatomyositis | 3 | 1.09 | 0.20 | 3.22 |  | 21 | 1.26 | 0.78 | 1.92 |  |
| Primary biliary cirrhosis | 0 |  |  |  |  | 16 | 1.28 | 0.73 | 2.09 |  |
| Psoriasis | 157 | **1.56** | **1.33** | **1.83** |  | 367 | **1.53** | **1.38** | **1.70** |  |
| Reiter´s disease | 5 | 2.38 | 0.75 | 5.60 |  | 8 | 1.26 | 0.54 | 2.49 |  |
| Rheumatic fever | 12 | **2.39** | **1.23** | **4.19** |  | 19 | **2.00** | **1.20** | **3.13** |  |
| Rheumatoid arthritis | 648 | **1.68** | **1.55** | **1.82** |  | 1607 | **1.48** | **1.41** | **1.55** |  |
| Sarcoidosis | 56 | **1.64** | **1.24** | **2.13** |  | 121 | 1.08 | 0.89 | 1.29 |  |
| Sjögren´s syndrome | 12 | 1.38 | 0.71 | 2.41 |  | 46 | 1.17 | 0.86 | 1.57 |  |
| Systemic lupus erythematosus | 50 | **2.34** | **1.74** | **3.09** |  | 122 | **1.78** | **1.48** | **2.13** |  |
| Systemic sclerosis | 11 | 0.86 | 0.43 | 1.55 |  | 30 | 1.22 | 0.83 | 1.75 |  |
| Ulcerative colitis | 104 | 1.06 | 0.86 | 1.28 |  | 435 | **1.17** | **1.07** | **1.29** |  |
| Wegener´s granulomatosis | 7 | 0.64 | 0.26 | 1.34 |  | 43 | 1.11 | 0.81 | 1.50 |  |
| All | 2442 | **1.51** | **1.45** | **1.57** |  | 5920 | **1.38** | **1.34** | **1.41** |  |
| O = observed number of cases; SIR = standardized incidence ratio; CI = confidence interval. | | | | | | | | | |  |
| Bold type: 95% CI does not include 1.00. |  |  |  |  |  |  |  |  |  |  |
| Adjusted for age, period, socioeconomic status, region of residence, hospitalization of chronic lower respiratory diseases, obesity, alcoholism, hypertension, diabetes, atrial fibrillation, heart failure, renal disease, sepsis, and coronary heart disease. | | | | | | | | | | |
